# Design, Synthesis, and Biological Evaluation of Potent and Selective Inhibitors of Ataxia Telangiectasia Mutated and Rad3-Related (ATR) Kinase for the Efficient Treatment of Cancer

**DOI:** 10.3390/molecules28114521

**Published:** 2023-06-02

**Authors:** Jialu Shao, Lei Huang, Wenwen Lai, Yi Zou, Qihua Zhu

**Affiliations:** 1 Department of Medicinal Chemistry, School of Pharmacy, China Pharmaceutical University, Nanjing 210009, China; shaojialu1997@126.com (J.S.); huangleiphd2019@163.com (L.H.); laiwenw@126.com (W.L.); 2 Department of Pharmacology and Medicinal Chemistry, Jiangsu Vocational College of Medicine, Yancheng 224005, China; 3 Jiangsu Key Laboratory of Drug Design and Optimization, Department of Medicinal Chemistry, China Pharmaceutical University, Nanjing 211198, China

**Keywords:** ATR, ATR inhibitors, ATM, DDR, synthetic lethality, cancer

## Abstract

Ataxia telangiectasia mutated and Rad3-related (ATR), a vital member of the phosphatidylinositol 3-kinase-related kinase (PIKK) family, plays a critical role in the DNA damage response (DDR). Tumor cells with a loss of DDR function or defects in the ataxia telangiectasia mutated (ATM) gene are generally more dependent on ATR for survival, suggesting that ATR is an attractive anticancer drug target based on its synthetic lethality. Herein, we present a potent and highly selective ATR inhibitor, **ZH-12** (IC_50_ = 0.0068 μM). It showed potent antitumor activity as a single agent or in combination with cisplatin in the human colorectal adenocarcinoma LoVo tumor xenograft mouse model. Overall, **ZH-12** may be a promising ATR inhibitor based on the principle of synthetic lethality and deserves further in-depth study.

## 1. Introduction

DNA damage response (DDR) is a highly conserved intracellular defense mechanism against DNA damage induced by external and intrinsic factors [1,2]. The DDR comprises DNA repair and cell checkpoint pathways for detecting and repairing DNA damage [3]. Central to the DDR is ataxia telangiectasia mutated and Rad3-related (ATR) and ataxia telangiectasia mutated kinase (ATM), which induce cell cycle arrest and facilitate DNA repair [4,5,6]. ATR and ATM belong to the phosphatidylinositol 3-kinase-related kinase (PIKK) family of serine/threonine protein kinases, which also comprises DNA-dependent protein kinase (DNA-PK) and the mammalian target of rapamycin (mTOR) [7]. Although ATR and ATM are known to share structural similarities, they are primarily activated and respond to different types of DNA damage. ATR is mainly implicated in the repair of single-stranded DNA breaks (SSB), whereas ATM responds to double-stranded DNA breaks (DSB) [8,9].

However, it has been found that ATM function is often impaired in tumor cells (from defects in expression, activation, or downstream signaling proteins), resulting in genomic instability, which is advantageous to tumor development [10,11]. Therefore, cells harboring impaired ATM function will depend more on ATR to maintain survival, suggesting ATR and ATM are synthetically lethal gene pairs. Many preclinical studies have validated that cancer cells with ATM signaling defects are susceptible to ATR inhibition after being treated with DNA-damaging drugs [12,13]. In addition, normal cells can tolerate ATR inhibition due to the presence of ATM-mediated compensatory DDR [14,15]. Therefore, targeting ATR may be a promising strategy to treat cancer-harbored defects in ATM signaling, attracting broad interest in industry and academia.

**Figure 1:**
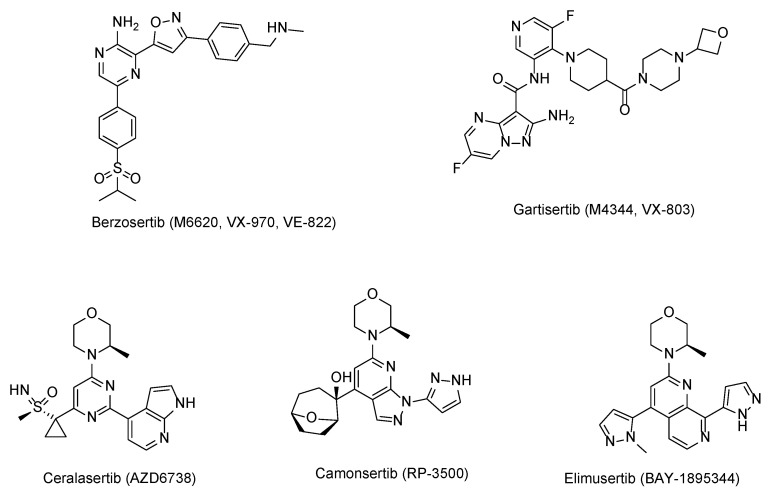
Structures of the ATR inhibitors in clinical trials.

To date, five selective ATR inhibitors (berzosertib [16], ceralasertib [17], M4344 [18], RP-3500 [19], and BAY-1895344 [20]) have been forwarded to clinical trials to evaluate their effects on cancer treatment as a single agent or combination therapy (Figure 1). All of the compounds were shown to modulate ATR/CHK1 signaling in an ATR-dependent manner and promote the progression of the G2/M phase, resulting in apoptosis without repair of the damaged DNA. Among them, ceralasertib (AZD6738), the oral ATR inhibitor, has been used in clinical trials as both monotherapy and combination therapy strategies, including chronic lymphocytic leukemia (CLL) [21], mantle cell lymphoma [22], gastric cancer [23], and breast cancer [24]. Although these ATR inhibitors have shown promising efficacy in clinical studies, none is available on the market. Therefore, it is still necessary to widen the diversity of the ATR inhibitors.

Herein, we describe the design, synthesis, and biological evaluation of a series of ATR inhibitors. Among them, **ZH-12** shows potent activity against ATR with an IC_50_ value of 0.0068 μM with high selectivity. Furthermore, **ZH-12** showed potent antitumor efficacy in vivo as a single agent or in combination with cisplatin.

## 2. Results and Discussion

### 2.1. Design of ATR Inhibitors

**Figure 2:**
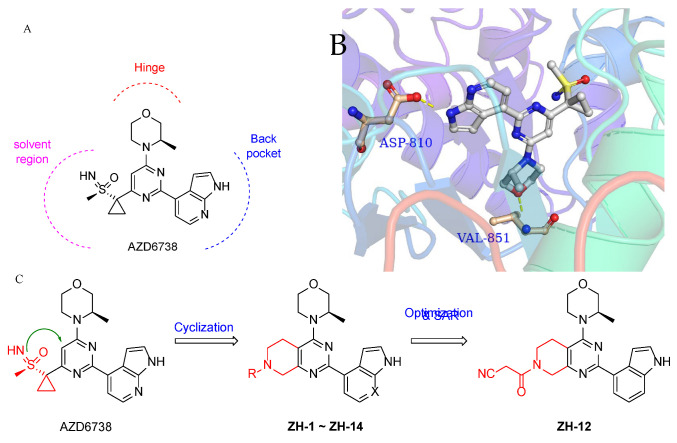
Design of ATR inhibitors. (**A**) Structure of AZD6738. (**B**) Molecular docking of AZD6738 with a PI3Kα mutant mimicking ATR (PDB: 5UK8). (**C**) The design strategy of the ATR inhibitors.

We chose AZD6738 as the lead compound and tried to modify the structure utilizing the structure-based drug design (SBDD) strategy. Firstly, a docking study was performed. Due to ATR having high homology with PI3Kα, PI3Kα-mutants were used to mimic the ATP binding site of ATR [25]. AZD6738 was docked into the PI3Kα-mutants (PDB: 5UK8) (Figure 2A,B) [25], and the results demonstrated that the (*R*)-3-methyl morpholinyl group of AZD6738 formed a critical hydrogen bond with VAL-851 in the hinge region. The NH-group of azaindole in AZD6738 also formed a hydrogen bond with ASP-810, which plays a crucial role in the binding of ATR. The sulfoxide imine group of AZD6738 was extended to the solvent region, which does not have direct interactions with residues in the active site, providing a potential site for our structural modification (Figure 2A,B). In addition, we found a cavity between sulfoxide imine and the pyrimidine scaffold, which could be filled using a particular group.

Based on the above observations, we designed the 5,6,7,8-tetrahydropyrido[3,4-*d*]pyrimidin-4-yl scaffold using a cyclization strategy (Figure 2C). As a result, a series of 5,6,7,8-tetrahydropyrido[3,4-*d*]pyrimidin-4-yl derivatives (**ZH-1**~**ZH-14**) were synthesized, and their activities against the ATR enzyme in vitro were evaluated. All the designed compounds were reasonable because their clogPs were < 5 and complied with the rule of clogP within the rule of five. As shown in Table 1, **ZH-1** with ethyl sulfonyl at 7-position of 5,6,7,8-tetrahydropyrido[3,4-*d*]pyrimidin-4-yl scaffold exhibited moderate ATR inhibition (IC_50_ = 53.26 nM), which was less potent than AZD6738 (IC_50_ = 6.50 nM). However, this result provided a guide for further structural modification. Subsequently, the acyl group moieties were introduced at the 7-position to mimic the sulfoxide imine group in AZD6738 to obtain more potent ATR inhibitors. Among them, propionamide **ZH-2**, butyramide **ZH-3,** and 3-methylbutanamide **ZH-4** exhibited a comparable potency for ATR to compound **ZH-1,** suggesting the acyl moiety warranted further investigation. Compound **ZH-5** was obtained by introducing the 2-cyanoacetyl group, and its ATR activity (IC_50_ = 11.34 nM) was significantly improved. However, the cyano group in **ZH-5** was replaced with a hydroxyl group to give compound **ZH-6** (IC_50_ = 34.03 nM), which was less potent than **ZH-4**, suggesting the polar group was not tolerated at this site. As expected, we replaced the hydroxyl group in **ZH-6** with a methoxyl group to give compound **ZH-7**, and its ATR activity was significantly improved. Then, some cycloalkyl groups were introduced to the 7-position to afford compounds **ZH-8**~**ZH-10**. These compounds exhibited good ATR inhibitory activities. Compound **ZH-10** (IC_50_ = 27.47 nM) showed a comparable ATR inhibitory activity to compound **ZH-5**. In addition, butyramide was replaced with ethylurea to afford compound **ZH-7**, which exhibited comparable ATR activity to compounds **ZH-5** and **ZH-10**. It was identified that the indole group could retain the ATR potency of the 7-azaindole. Therefore, we replaced the 7-azaindole group in compounds **ZH-5**, **ZH-7**, and **ZH-11** with the indole group to give **ZH-12**~**ZH-13,** respectively. As expected, these compounds exhibited good ATR inhibitory activities. Compound **ZH-12** showed a single-digit nanomole inhibitory activity (IC_50_ = 6.81 nM) comparable to AZD6738.

### 2.2. Chemistry

**Scheme 1:**
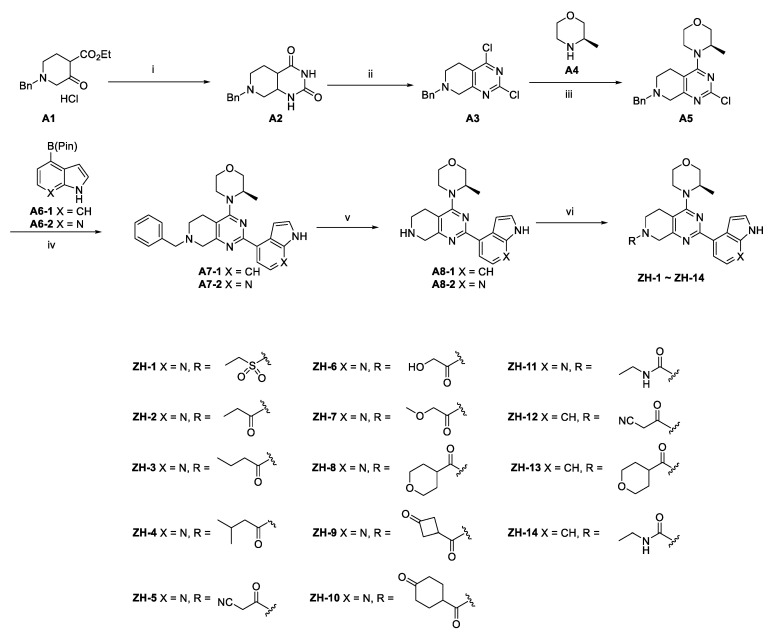
Reagents and reaction conditions: (i) NH_2_CONH_2_, MeONa, MeOH, 0 °C to reflux, 8 h, 83.1% yield; (ii) POCl_3_, 0 °C to reflux, 12 h, 71.5% yield; (iii) **A4**, Na_2_CO_3_, MeCN, rt to 120 °C, 12 h, 64.9% yield; (iv) **A6-1** or **A6-2**, Pd(Ph_3_)_2_Cl_2_, Na_2_CO_3_, EtOH, reflux, 12 h, 76.3–81.5% yield; (v) H_2_, 10% Pd/C, HCOOH/H_2_O (1/5), 30 °C, 6 h, 75.4–80.8% yield; (vi) **ZH-1**~**ZH-4**, **ZH-8,** and **ZH-13**: **A8**, corresponding sulfonyl chloride or acyl chloride, Et_3_N, DCM, rt, 1 h, 61.5–84.1% yield; **ZH-5**~**ZH-7**, **ZH-9**, **ZH-10,** and **ZH-12**: corresponding carboxylic acid, EDCI, HOBT, DCM, rt, 1 h, then **A8**, Et_3_N, DCM, rt, 4 h, 55.4–79.6% yield; **ZH-11**, **ZH-14**: **A8**, corresponding alkyl isocyanate, DCM, 0 °C to rt, 4 h, 78.1–88.0% yield.

The synthetic routes of the target compounds and intermediates are outlined in Scheme 1. In brief, pinner pyrimidine synthesis was carried out to afford the intermediate **A2** with ethyl *N*-benzyl-3-oxo-4-piperidine-carboxylate hydrochloride (**A1**) and urea in the presence of MeONa. Then, chlorination of the intermediate **A2** with the POCl_3_ generated the intermediate **A3**, which was followed by nucleophilic substitution with (*R*)-3-methyl morpholine (**A4**) to provide the intermediate **A5**. Compound **A5** underwent a palladium-catalyzed Suzuki coupling reaction with **A6** to afford the intermediate **A7**. The **A8** was obtained by debenzylation in formic acid and H_2_O (1:5) solution with Pd/C as a catalyst under atmospheric hydrogen pressure. Finally, **A8** was reacted with a corresponding sulfonyl chloride, acyl chloride, or alkyl isocyanate to generate the target compounds **ZH-1**~**ZH-4**, **ZH-8**, **ZH-11**, **ZH-13**, and **ZH-14**. In addition, **ZH-5**~**ZH-7**, **ZH-9**, **ZH-10,** and **ZH-12** were generated by carboxylic acid reacted with **A8** in the presence of EDCI and HOBT.

### 2.3. Molecular Docking of ***ZH-12***

**Figure 3:**
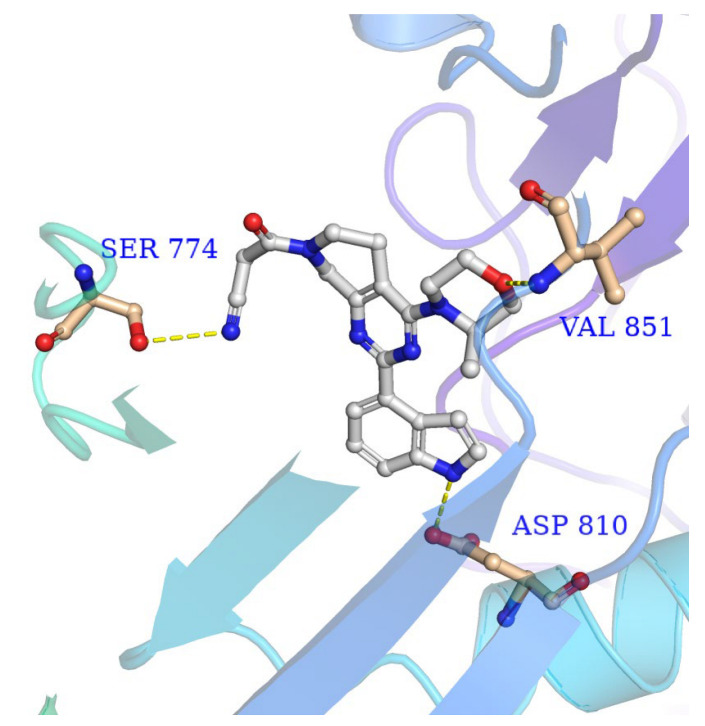
The binding mode of **ZH-12** with PI3Kα-mutants mimicking ATR (PDB: 5UK8).

Molecular docking was performed to predict the binding mode of the **ZH-12** with ATR kinase. **ZH-12** was docked into the ATR protein homology model based on the PI3Kα-mutants (PDB: 5UK8). As shown in Figure 3, **ZH-12** bound to the active site of the PI3Kα-mutants protein, similar to the AZD6738. The 3-methyl morpholine group formed a critical hydrogen bond with residue VAL-851 at the kinase hinge binder. In addition, the indole group formed a hydrogen bond with residue ASP-810. Finally, the cyanoacetyl group formed a hydrogen bond with residue SER-774.

### 2.4. Antiproliferative Activity In Vitro

Based on the above results, **ZH-4**, **ZH-7**, and **ZH-12** were selected to evaluate their antiproliferative activities using the CCK-8 assay. Four tumor cell lines were chosen, including two ATM-deficient cell lines, LoVo and NCI-H23, and two ATM-normal cell lines, HCT116 and A549 [23,26,27]. As shown in Table 2, compounds **ZH-7** and **ZH-12** exerted promising antiproliferative activity on LoVo and NCI-H23, which had higher antiproliferative activity than A549 and HCT116. Compared to **ZH-7** and **ZH-12**, **ZH-4** exhibited a reversed result in that it had more potent antiproliferation activities against the HCT116 rather than the LoVo cell lines, probably because **ZH-4** also inhibits other kinases in the PIKK family in HCT116 cells, resulting in off-target effects. To our delight, **ZH-12** showed comparable antitumor activity to the positive drug AZD6738 against LoVo, NCI-H23, HCT116, and A549 cells, with IC_50_ values of 0.29, 0.77, 1.61, and 4.74 μM respectively. Therefore, **ZH-12** has excellent potency for cell viability inhibition in ATM-deficient cell lines and warrants further pharmacological studies.

**Figure 4:**
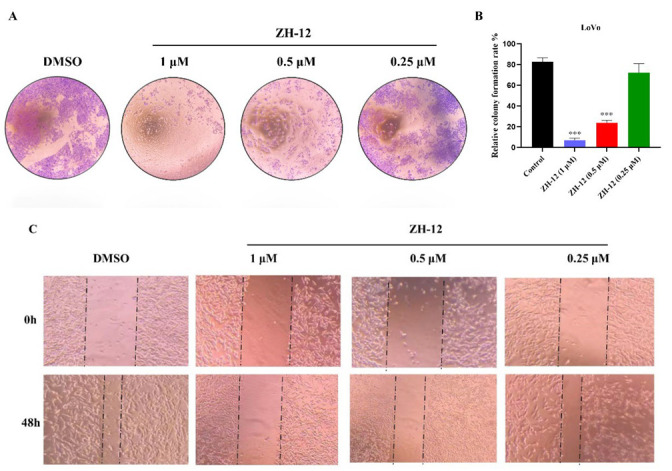
Antitumor activities of **ZH-12** in vitro. (**A**) Colony formation assays in LoVo cells. Cells were incubated with various concentrations of **ZH-12** for 24 h; then, the medium was refreshed with a drug-free medium every two days. (**B**) On day 14, the cells were stained with crystal violet and quantified. (**C**) In vitro antimigratory activity of **ZH-12** against LoVo cells. Cells were treated with different concentrations of **ZH-12** for 48 h. The bar graphs present the mean ± SD. Statistical analysis was performed by one-way analysis of variance (ANOVA). *n* = 3, *** *p* < 0.001 vs. the control group.

We then examined the ability of **ZH-12** to inhibit tumor cell proliferation in LoVo cells using a colony-forming assay. As shown in Figure 4, **ZH-12** inhibited cell proliferation dose-dependently (Figure 4A,B). In addition, the wound healing assays showed that **ZH-12** also inhibited LoVo cell migration in a dose-dependent manner (Figure 4C). In conclusion, we have identified a candidate compound **ZH-12**, which significantly inhibits tumor cell proliferation and migration.

### 2.5. Cellular Mechanism of Action Studies

#### 2.5.1. Comet Assay

**Figure 5:**
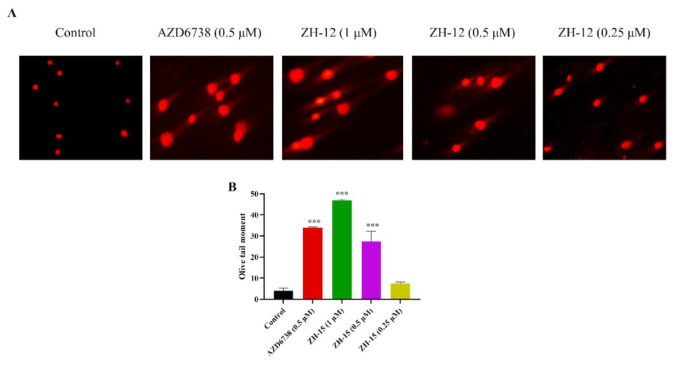
Effects of **ZH-12** and AZD6738 on the DNA damage response in LoVo cells. (**A**) The LoVo cancer cells were treated with the indicated compound for 72 h. Comet images ×200 taken by fluorescence microscope are shown. (**B**) Quantitative analysis was performed with the comet analysis software CASP, and the olive tail moment was employed to evaluate the DNA damage. The bar graphs present the mean ± SD. Statistical analysis was performed by one-way analysis of variance (ANOVA). *n* = 3, *** *p* < 0.001 vs. the control group.

ATR is a significant member of the DNA damage checkpoint [28]. A comet assay was conducted to further evaluate the extent of the DNA damage induced by **ZH-12**. As shown in Figure 5, **ZH-12** induced significant amounts of DNA damage dose-dependently. It generated a comparable tail intensity to AZD6738 at 0.5 µM.

#### 2.5.2. Cell Apoptosis Assay

**Figure 6:**
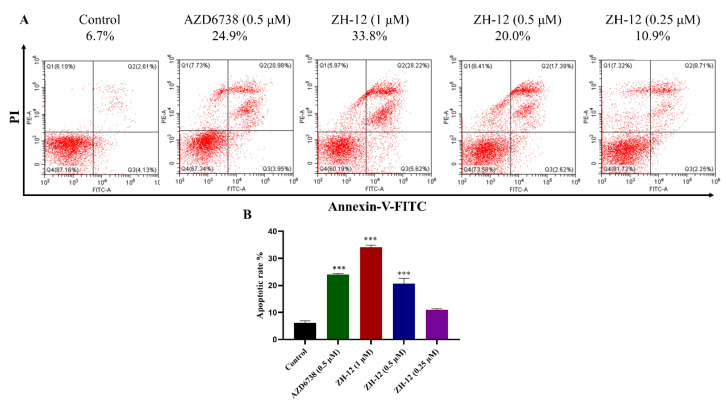
Apoptosis assay of **ZH-12** in LoVo cells. (**A**) Apoptotic index analysis of LoVo cells detected by Annexin V-fluorescein isothiocyanate isomer I (FITC)/propidium iodide (PI) staining after treatment with **ZH-12**. (**B**) The percentage of apoptotic cells was counted by GraphPad Prism 8.0. The bar graphs present the mean ± SD. Statistical analysis was performed by one-way analysis of variance (ANOVA). *n* = 3, *** *p* < 0.001 vs. the control group.

An apoptosis assay was conducted using Annexin-V by FACS analysis in the LoVo cells to determine the effect of **ZH-12** on cell death. As shown in Figure 6, **ZH-12** can induce cell apoptosis dose-dependently. In addition, the ability of **ZH-12** (0.5 μM) to induce cell apoptosis was comparable to AZD6738 (0.5 μM). Notably, the apoptosis effect of **ZH-12** was achieved 33.8% at 1 μM (Figure 6A,B). The above results showed that compound **ZH-12** could significantly induce apoptosis of tumor cells, thereby inhibiting the growth of LoVo cells.

#### 2.5.3. Western Blot Analysis of Protein Expression In Vitro

**Figure 7:**
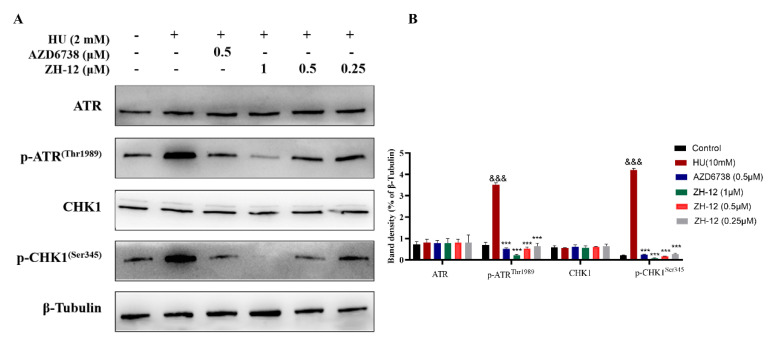
**ZH-12** inhibited the phosphorylation of ATR and CHK1 in LoVo cells in a dose-dependent manner. (**A**) Western blot analyses of ATR, p-ATR^(Thr1989)^, CHK1, and p-CHK1^(Ser345)^ protein expression in LoVo cells treated with the indicated doses of **ZH-12** and HU (hydroxyurea). (**B**) Band intensity of ATR, p-ATR^(Thr1989)^, CHK1, and p-Chk1^(Ser345)^ relative to the control in LoVo cells. The bar graphs present the mean ± SD. Statistical analysis was performed by one-way analysis of variance (ANOVA). *n* = 3, *** *p* < 0.001 and ^&&&^ *p* < 0.001 vs. the control group.

To verify the mechanism of action of compound **ZH-12** causing DNA damage, we further investigated the effect of the ATR signaling pathways in intact cells by Western blot assays. Firstly, LoVo (ATM-deficient) cells were treated with HU (hydroxyurea) to stimulate DNA damage. As shown in Figure 7, **ZH-12** inhibited the phosphorylation of ATR and its downstream kinase CHK1 in a dose-dependent manner. At the same time, the ATR and CHK1 expression remained unchanged, indicating that **ZH-12** effectively inhibited the ATR signaling in intact cells (Figure 7A,B).

### 2.6. In Vivo Antitumor Effects of ***ZH-12***

**Figure 8:**
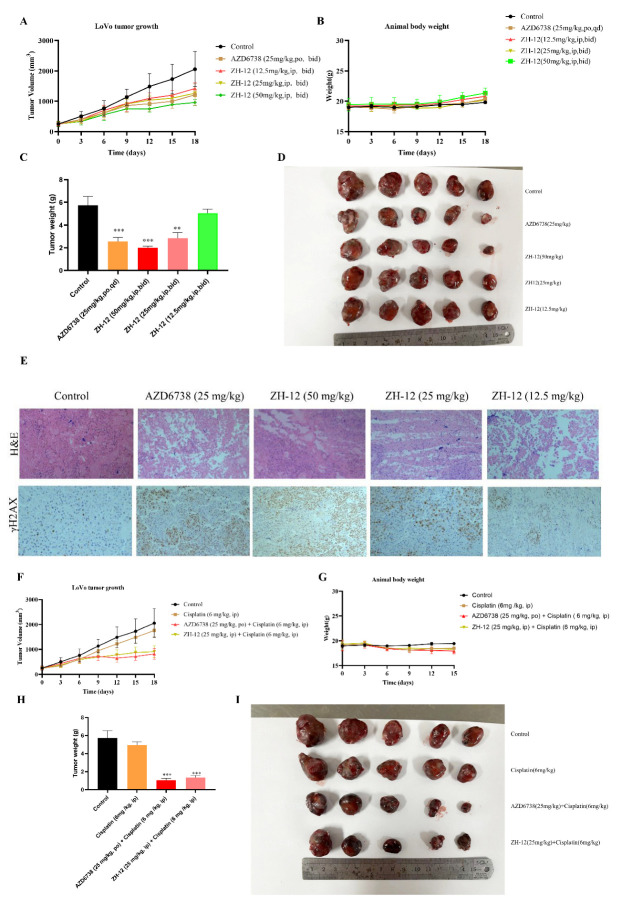
**ZH-12** inhibits the growth of LoVo xenografts and the expression of γH2AX in vivo. AZD6738 was administered orally, and **ZH-12** was injected intraperitoneally twice daily during days 1–18. (**A**) Tumor growth curve: AZD6738 (25 mg/kg, TGI: 42.5%), **ZH-12** (12.5 mg/kg, TGI: 27.0%), **ZH-12** (25 mg/kg, TGI: 39.5%), and **ZH-12** (50 mg/kg, 53.7%). (**B**) Body weight. (**C**) Tumor weight. (**D**) tumor nodules. (**E**) H&E staining and γH2AX expression of tumor sections. Scale bar, 50 μM. (**F**) Tumor growth curve: cisplatin (6 mg/kg, TGI: 26.1%), cisplatin+AZD6738 (6 + 25 mg/kg, TGI:66.4%), and cisplatin + **ZH-12** (6 + 25 mg/kg, TGI: 60.8%). (**G**) Body weight. (**H**) Tumor weight. (I) Tumor nodules. The bar graphs present the mean ± SD. Statistical analysis was performed by one-way ANOVA. *n* = 3, ** *p* < 0.01, *** *p* < 0.001 vs. the control group.

Finally, we evaluated the antitumor activity of **ZH-12** in the human colorectal adenocarcinoma LoVo tumor xenograft mouse model as a single agent or in combination with cytotoxic agents. Firstly, compound **ZH-12** was administered at 12.5, 25, and 50 mg/kg twice a day (BID) for 18 consecutive days to assess its antitumor activity as a single agent in vivo, and the AZD6738 (25 mg/kg) was chosen as a positive drug. As shown in Figure 8, **ZH-12** significantly inhibited the growth of LoVo xenografts in a dose-dependent manner. At 25 mg/kg dosage, **ZH-12** showed comparable tumor growth inhibition (TGI) to that of the positive drug AZD6738 (25 mg/kg). Notably, **ZH-12** (50 mg/kg) inhibited tumor growth with a TGI of 53.8% (Figure 8A,C,D). No significant body weight loss or toxicity was observed during the process (Figure 8B). Subsequently, HE staining and immunohistochemistry (IHC) analysis were performed to measure the effect of different doses of **ZH-12** on tumor histomorphology and DNA repair (γH2AX, a DNA damage biomarker) in LoVo tumor xenograft models. As shown in Figure 8, **ZH-12** significantly altered the morphologic features of the tumor tissue compared to the control group, including cell shrinkage, condensation, and nuclear chromatin marginalization (Figure 8E). γH2AX levels decreased dose-dependently, suggesting that the ATR-mediated DNA damage was promoted in tumor tissue (Figure 8E). The above results indicated that compound **ZH-12** suppressed tumor growth in vivo by promoting DNA damage without toxicity.

Most ATR inhibitors are combined with various genotoxic chemotherapeutic agents (e.g., cisplatin and cyclophosphamide), which have shown strong synergistic effects [29,30]. Therefore, to evaluate the antitumor activity of **ZH-12** in combination with cisplatin in vivo, we investigated the efficacy of cisplatin (6 mg/kg, twice a week) alone or in combination with **ZH-12** (25 mg/kg, once daily) or AZD6738 (25 mg/kg, once daily) in a colorectal LoVo xenograft balb/c nude mice model. The results showed that cisplatin alone could inhibit tumor growth, and its efficacy was significantly improved when combined with AZD6738 (Figure 8F,H,I). To our delight, cisplatin combined with **ZH-12** also considerably inhibited tumor regrowth with a TGI value of 60.8%, demonstrating comparable antitumor efficacy to the group of AZD6738 combined with cisplatin. We examined the combinatorial toxicity by weight loss, and the results showed that cisplatin and the combined treatment resulted in comparable maximum average body weight loss, indicating that the toxicity may be ascribed to cisplatin (Figure 8G). These results revealed that **ZH-12** has the advantages of high efficacy and low toxicity when combined with cisplatin treatment.

### 2.7. In Vivo Western Blot Analysis

**Figure 9:**
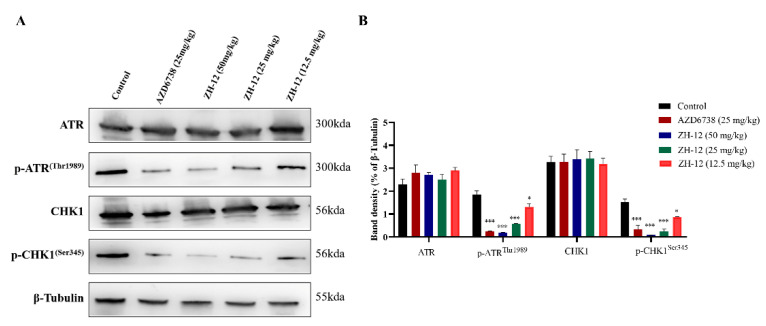
Effect of **ZH-12** on ATR/CHK1 signaling in vivo. (**A**) Western blot analysis of ATR, p-ATR^Thr1989^, p-CHK1, and p-Chk1^Ser345^ protein expression in tumor tissues from LoVo tumor xenografts treated with the 50, 25, and 12.5 mg/kg doses of **ZH-12** or vehicle. (**B**) The intensity of the ATR, p-ATR^Thr1989^, CHK1, and p-CHK1^Ser345^ was quantified by ImageJ and normalized to the β-tubulin. The bar graphs present the mean ± SD. Statistical analysis was performed by one-way ANOVA. *n* = 3, *** *p* < 0.001, * *p* < 0.05 vs. the control group.

To further investigate the effect of compound **ZH-12** on the ATR/CHK1 signaling pathway in vivo, the tumors were excised and subjected to Western blot analysis. The ATR/CHK1 signaling pathway plays an essential role in antitumor effects by regulating cycle checkpoints and DNA damage repair. As shown in Figure 9, **ZH-12** significantly inhibited ATR phosphorylation (p-ATR^Thr1989^) and CHK1 phosphorylation (p-CHK1^Ser345^) in a dose-dependent manner compared to the control group. Still, it did not affect the expression level of ATR and CHK1, suggesting that the ATR-mediated downstream signaling pathways were impaired (Figure 9A,B). These results indicate that **ZH-12** suppresses LoVo xenograft tumor growth in vivo by inhibiting the ATR/CHK1 signaling pathway.

### 2.8. Kinase Selectivity of the Most Potent Compound ***ZH-12***

**Figure 10:**
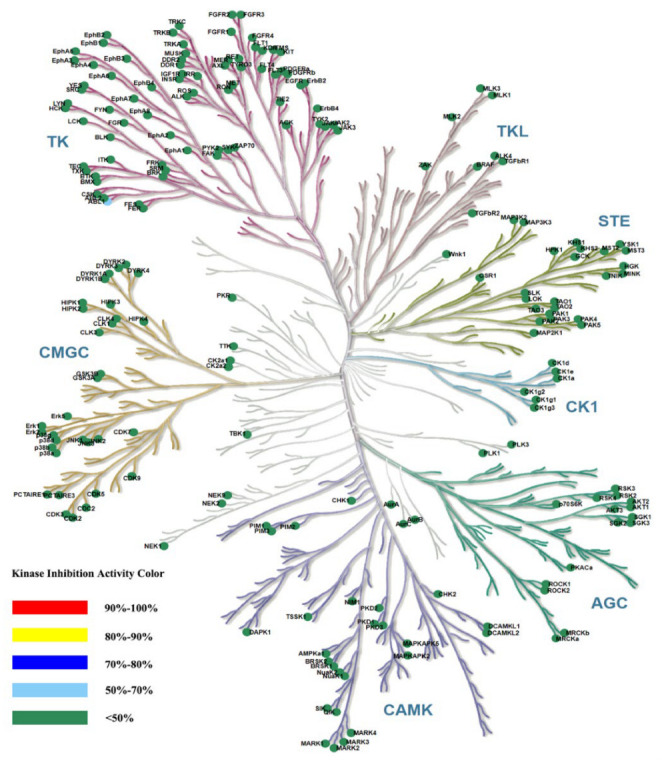
A kinase panel screen showed the kinase selectivity profile of compound **ZH-12** against 207 kinases assayed at 1 μM in duplicate of two independent experiments. Compound selectivity is represented in a dendrogram view of the human kinome phylogenetic tree. The color code for inhibition is indicated.

Compound **ZH-12** was tested at a single concentration of 1 μM against the Ion Channel Explorer kinase panel to further elucidate its kinase selectivity. The results showed that **ZH-12** at a concentration of 1 μM displayed weak or no inhibitory activities against 207 kinases (Figure 10), demonstrating the high selectivity of **ZH-12** for ATR. These results suggest that **ZH-12** has excellent kinase selectivity for ATR over other human protein kinases.

## 3. Experiments

### 3.1. General Chemistry

All reagents and solvents were purchased from chemical suppliers and used without further purification. All reactions were monitored by TLC on silica gel plates (silica gel 60 F_254_) with UV (254 or 365 nm). ^1^H NMR (300 or 400 MHz) and ^13^C NMR (100 MHz) spectra were recorded on Bruker spectrometers with TMS as the internal standard and the chemical shift values (*δ*) expressed in ppm. Coupling constants (*J*) are described in hertz (Hz). Ordinary and high-resolution mass spectra were recorded by ESI-MS. The purity of the target compounds was >90%, determined by HPLC (BDS Hypersil C18, λ = 254 nm). The synthesis conditions of the intermediates **A2**~**A8** and the ^1^H NMR, ^13^C NMR, and HRMS spectra of the target compounds are shown in the Supplementary Files.

#### 3.1.1. General Procedure for the Synthesis of **ZH-1**~**ZH-4**, **ZH-8**, and **ZH-13**

Ethyl sulfonyl chloride or appropriate acyl chloride (0.430 mmol) was dropped into a solution of **A8-2** (0.286 mmol) in DCM (5 mL) followed by Et_3_N (0.572 mmol). After stirring at room temperature for 1 h, H_2_O (5 mL) was added, and the mixture was extracted with DCM (3 × 5 mL). The combined organic layers were dried with Na_2_SO_4_, filtered, and concentrated under reduced pressure. The residue was purified by column chromatography on silica gel to give the compounds **ZH-1**~**ZH-4**, **ZH-8**, and **ZH-13**.

*(R)-4-(7-(ethylsulfonyl)-2-(1H-pyrrolo[2,3-b]pyridin-4-yl)-5,6,7,8-tetrahydropyrido[3,4-d]pyrimidin-4-yl)-3-methylmorpholine* (**ZH-1**). White solid (84 mg). Yield 66.5%, m.p. 244–246 °C. ^1^H NMR (400 MHz, DMSO-*d*_6_) *δ* (ppm): 11.78 (s, 1H), 8.34 (d, *J* = 5.0 Hz, 1H), 7.97 (d, *J* = 5.0 Hz, 1H), 7.58 (t, *J* = 3.0 Hz, 1H), 7.28 (dd, *J* = 3.4, 2.0 Hz, 1H), 4.55–4.44 (m, 2H), 4.25–4.14 (m, 1H), 3.94–3.91 (m, 1H), 3.77–3.73 (m, 1H), 3.76–3.70 (m, 1H), 3.67–3.65 (m, 2H), 3.62–3.54 (m, 1H), 3.52–3.51 (m, 1H), 3.45–3.38 (m, 1H), 3.25 (q, *J* = 7.4 Hz, 2H), 2.85–2.79 (m, 2H), 1.31–1.21 (m, 6H). ^13^C NMR (101 MHz, DMSO-*d*_6_) *δ* (ppm): 164.71, 160.81, 160.64, 150.66, 142.73, 136.62, 127.65, 118.14, 114.86, 114.47, 102.37, 70.86, 66.85, 50.22, 49.52, 43.94, 43.03, 26.91, 14.65, 8.09. HRMS (ESI): *m*/*z* calcd for C_21_H_27_N_6_O_3_S: 443.1865 [M + H]^+^; found: 443.1858. HPLC purity = 96.70%, t_R_ = 3.346 min.

*(R)-1-(4-(3-methylmorpholino)-2-(1H-pyrrolo[2,3-b]pyridin-4-yl)-5,8-dihydropyrido[3,4-d]pyrimidin-7(6H)-yl)propan-1-one* (**ZH-2**). White solid (90 mg). Yield 77.6%, m.p. 234–236 °C. ^1^H NMR (300 MHz, CDCl_3_) *δ* (ppm): 10.42 (s, 1H), 8.46–8.44 (m, 1H), 8.12–8.07 (m, 1H), 7.53–7.36 (m, 2H), 5.17–4.59 (m, 2H), 4.19–4.09 (m, 1H), 4.02–4.00 (m, 1H), 3.89–3.49 (m, 7H), 2.82–2.71 (m, 2H), 2.51 (q, *J* = 7.3 Hz, 2H), 1.37 (d, *J* = 6.7 Hz, 3H), 1.30–1.18 (m, 3H). ^13^C NMR (101 MHz, CDCl_3_) *δ* (ppm): 172.80, 164.96, 162.17, 160.91, 150.36, 142.76, 137.65, 126.09, 118.53, 115.42, 114.77, 102.79, 71.10, 67.15, 50.54, 50.00, 43.06, 39.05, 27.04, 26.23, 14.55, 9.31. HRMS (ESI): *m*/*z* calcd for C_22_H_27_N_6_O_2_: 407.2195 [M + H]^+^; found: 407.2182. HPLC purity = 92.88%, t_R_ = 8.141 min.

*(R)-1-(4-(3-methylmorpholino)-2-(1H-pyrrolo[2,3-b]pyridin-4-yl)-5,8-dihydropyrido[3,4-d]pyrimidin-7(6H)-yl)butan-1-one* (**ZH-3**). White solid (110 mg). Yield 91.7%, m.p. 214–216 °C. ^1^H NMR (400 MHz, CDCl_3_) *δ* (ppm): 10.44 (s, 1H), 8.49–8.48 (m, 1H), 8.12–8.09 (m, 1H), 7.61–7.37 (m, 2H), 5.26–4.60 (m, 2H), 4.22–3.98 (m, 3H), 3.98–3.49 (m, 6H), 2.85–2.71 (m, 2H), 2.48 (q, *J* = 6.5 Hz, 2H), 1.85–1.70 (m, 2H), 1.39 (d, *J* = 6.7 Hz, 3H), 1.09–1.00 (m, 3H). ^13^C NMR (101 MHz, CDCl_3_) *δ* (ppm): 172.05, 164.95, 162.18, 160.95, 150.36, 142.77, 137.66, 126.08, 118.53, 115.43, 114.76, 102.80, 71.10, 67.15, 50.54, 47.34, 43.06, 39.00, 35.77, 26.26, 18.59, 14.55, 14.02. HRMS (ESI): *m*/*z* calcd for C_23_H_29_N_6_O_2_: 421.2352 [M + H]^+^; found: 421.2343. HPLC purity = 97.09%, t_R_ = 10.759 min.

*(R)-3-methyl-1-(4-(3-methylmorpholino)-2-(1H-pyrrolo[2,3-b]pyridin-4-yl)-5,8-dihydropyrido[3,4-d]pyrimidin-7(6H)-yl)butan-1-one* (**ZH-4**). White solid (108 mg). Yield 84.1%, m.p. 236–238 °C ^1^H NMR (300 MHz, CDCl_3_) *δ* (ppm): 10.54 (s, 1H), 8.45 (t, *J* = 4.8 Hz, 1H), 8.12–8.08 (m, 1H), 7.50 (t, *J* = 3.6 Hz, 1H), 7.47–7.38 (m, 1H), 5.22–4.68 (m, 2H), 4.20–4.07 (m, 1H), 4.08–4.00 (m, 1H), 3.91–3.52 (m, 7H), 2.74 (t, *J* = 5.7 Hz, 2H), 2.37 (t, *J* = 5.7 Hz, 2H), 2.33–2.14 (m, 1H), 1.38 (d, *J* = 6.7 Hz, 3H), 1.03 (t, *J* = 5.6 Hz, 6H). ^13^C NMR (101 MHz, CDCl_3_) *δ* (ppm): 171.56, 164.95, 162.19, 160.97, 150.32, 142.80, 137.68, 126.04, 118.60, 115.45, 114.74, 102.82, 71.11, 67.15, 50.67, 50.40, 43.05, 42.59, 39.04, 26.30, 25.70, 22.77, 14.54. HRMS (ESI): *m*/*z* calcd for C_24_H_31_N_6_O_2_: 435.2508 [M + H]^+^; found: 435.2493. HPLC purity = 98.83%, t_R_ = 12.333 min.

(*R)-(4-(3-methylmorpholino)-2-(1H-pyrrolo[2,3-b]pyridin-4-yl)-5,8-dihydropyrido[3,4-d]pyrimidin-7(6H)-yl)(tetrahydro-2H-pyran-4-yl)methanone* (**ZH-8**). White solid (92 mg). Yield 69.7%, m.p. >250 °C. ^1^H NMR (300 MHz, DMSO-*d*_6_) *δ* (ppm): 11.78 (s, 1H), 8.33 (d, *J* = 5.5 Hz, 1H), 7.97 (d, *J* = 5.0 Hz, 1H), 7.58 (s, 1H), 7.31–7308 (m, 1H), 4.96–4.50 (m, 2H), 4.18 (s, 1H), 3.97–3.81 (m, 4H), 3.78–3.60 (m, 5H), 3.57–3.38 (m, 3H), 3.17–2.95 (m, 1H), 2.81 (s, 1H), 2.69 (s, 1H), 1.72–1.57 (m, 4H), 1.29 (d, *J* = 6.9 Hz, 3H). ^13^C NMR (101 MHz, DMSO-*d*_6_) *δ* (ppm): 173.23, 164.45, 161.87, 160.70, 150.65, 142.73, 136.77, 127.62, 118.13, 114.89, 102.32, 70.86, 66.80, 50.24, 47.27, 42.88, 42.46, 36.96, 29.43, 29.24, 27.39, 14.68. HRMS (ESI): *m*/*z* calcd for C_25_H_31_N_6_O_3_: 463.2458 [M + H]^+^; found: 463.2444. HPLC purity = 97.16%, t_R_ = 6.954 min.

*(R)-(2-(1H-indol-4-yl)-4-(3-methylmorpholino)-5,8-dihydropyrido[3,4-d]pyrimidin-7(6H)-yl)(tetrahydro-2H-pyran-4-yl)methanone* (**ZH-13**). White solid (86 mg). Yield 61.5%, m.p. 170–172 °C. ^1^H NMR (400 MHz, DMSO-*d*_6_) *δ* (ppm): 11.24 (s, 1H), 8.10 (d, *J* = 7.2 Hz, 1H), 7.53 (d, *J* = 7.9 Hz, 1H), 7.50–7.32 (m, 2H), 7.19 (t, *J* = 7.8 Hz, 1H), 4.95–4.41 (m, 2H), 4.13 (s, 1H), 3.98–3.82 (m, 4H), 3.74 (d, *J* = 11.2 Hz, 1H), 3.70–3.59 (m, 4H), 3.56–3.39 (m, 3H), 3.17–2.99 (m, 1H), 2.83–2.76 (m, 1H), 2.67 (s, 1H), 1.74–1.59 (m, 4H), 1.27 (d, *J* = 6.5 Hz, 3H). ^13^C NMR (101 MHz, DMSO-*d*_6_) *δ* (ppm): 173.21, 164.46, 162.42, 161.56, 137.51, 129.49, 126.69, 126.54, 128.88, 120.80, 114.09, 113.57, 103.74, 70.91, 66.80, 50.22, 47.32, 43.07, 42.58, 36.98, 29.45, 29.25, 27.16, 14.56. HRMS (ESI): *m*/*z* calcd for C_26_H_32_N_5_O_3_: 462.2505 [M + H]^+^; found: 462.2494. HPLC purity = 97.36%, t_R_ = 15.010 min.

#### 3.1.2. General Procedure for the Synthesis of **ZH-5**~**ZH-7**, **ZH-9**~**ZH-10**, and **ZH-12**

EDCI (0.858 mmol) and HOBT (0.572 mmol) were added to a solution of the appropriate acid (0.572 mmol) in DCM (5 mL). After stirring at room temperature for 30 min, **A8** (0.286 mmol) and Et_3_N (40 mg, 0.572 mmol) were added to the mixture. The mixture was stirred at room temperature for 4 h. After completion (monitored by TLC), H_2_O (5 mL) was added, and the mixture was extracted with DCM (3 × 5 mL). The combined organic layers were dried with Na_2_SO_4_, filtered, and concentrated under reduced pressure. The residue was purified by column chromatography on silica gel to give the compounds **ZH-5**~**ZH-7**, **ZH-9**~**ZH-10**, and **ZH-12**.

*(R)-3-(4-(3-methylmorpholino)-2-(1H-pyrrolo[2,3-b]pyridinedin-4-yl)-5,8-dihydropyrido[3,4-d]pyrimidin-7(6H)-yl)-3-oxopropanenitrile* (**ZH-5**). White solid, 84 mg, 70.5% yield, m.p. > 250 °C. ^1^H NMR (400 MHz, DMSO-*d*_6_) *δ* (ppm): 11.79 (s, 1H), 8.34 (d, *J* = 4.9 Hz, 1H), 7.97 (t, *J* = 4.8 Hz, 1H), 7.64–7.56 (m, 1H), 7.33–7.25 (m, 1H), 4.90–4.44 (m, 2H), 4.29–4.21 (m, 2H), 4.19–4.11 (m, 1H), 3.99–3.89 (m, 1H), 3.79–3.60 (m, 5H), 3.58–3.45 (m, 2H), 2.89–2.78 (m, 1H), 2.72 (t, *J* = 5.6 Hz, 1H), 1.32–1.21 (m, 3H). ^13^C NMR (101 MHz, DMSO-*d*_6_) *δ* (ppm): 164.56, 162.52, 161.21, 160.70, 150.66, 142.74, 136.64, 127.66, 118.14, 116.56, 114.89, 114.76, 102.34, 70.82, 66.85, 50.31, 49.78, 47.40, 42.94, 26.42, 25.40, 14.59. HRMS (ESI): *m*/*z* calcd for C_22_H_24_N_7_O_2_: 418.1991 [M + H]^+^; found: 418.1982. HPLC purity = 99.15%, t_R_ = 15.907 min.

*(R)-2-hydroxy-1-(4-(3-methylmorpholino)-2-(1H-pyrrolo[2,3-b]pyridinedin-4-yl)-5,8-dihydropyrido[3,4-d]pyrimidin-7(6H)-yl)ethan-1-one* (**ZH-6**). White solid (80 mg). Yield 68.6%, m.p. 174–176 °C. ^1^H NMR (400 MHz, DMSO-*d*_6_) *δ* (ppm): 11.78 (s, 1H), 8.34 (d, *J* = 5.0 Hz, 1H), 8.01–7.94 (m, 1H), 7.59 (s, 1H), 7.29 (s, 1H), 4.86–4.46 (m, 3H), 4.26 (d, *J* = 5.7 Hz, 1H), 4.16 (s, 1H), 3.94–3.91 (m, 1H), 3.79–3.59 (m, 5H), 3.54–3.50 (m, 2H), 2.80 (s, 1H), 2.72 (s, 1H), 1.28 (d, *J* = 6.7 Hz, 3H), 1.23 (s, 1H). ^13^C NMR (101 MHz, DMSO-*d*_6_) *δ* (ppm): 170.93, 164.51, 161.51, 160.67, 150.66, 142.73, 136.72, 127.63, 118.13, 114.88, 102.36, 70.83, 66.84, 60.88, 50.26, 47.14, 42.95, 38.85, 29.47, 26.69, 14.62. HRMS (ESI): *m*/*z* calcd for C_21_H_25_N_6_O_3_: 409.1988 [M + H]^+^; found: 409.1982. HPLC purity = 95.30%, t_R_ = 4.802 min.

*(R)-2-methoxy-1-(4-(3-methylmorpholino)-2-(1H-pyrrolo[2,3-b]pyridinedin-4-yl)-5,8-dihydropyrido[3,4-d]pyrimidin-7(6H)-yl)ethan-1-one* (**ZH-7**). White solid (96 mg) Yield 79.6%, m.p. 294–296 °C. ^1^H NMR (400 MHz, DMSO-*d*_6_) *δ* (ppm): 11.78 (s, 1H), 8.34 (d, *J* = 5.0 Hz, 1H), 7.98–7.97 (m, 1H), 7.59–7.58 (m, 1H), 7.36–7.25 (m, 1H), 4.86–4.48 (m, 2H), 4.27–4.24 (m, 2H), 4.21–4.10 (m, 1H), 3.98–3.89 (m, 1H), 3.78–3.59 (m, 5H), 3.58–3.46 (m, 2H), 3.36 (s, 3H), 2.80 (s, 1H), 2.71 (s, 1H), 1.28 (d, *J* = 6.6 Hz, 3H). ^13^C NMR (101 MHz, DMSO-*d*_6_) *δ* (ppm): 168.14, 164.49, 161.54, 160.69, 150.66, 142.73, 136.73, 127.63, 118.13, 114.90, 102.34, 99.88, 70.83, 66.83, 58.94, 50.24, 48.78, 46.95, 42.95, 38.62, 26.79, 14.64. HRMS (ESI): *m*/*z* calcd for C_22_H_27_N_6_O_3_: 423.2145 [M + H]^+^; found: 423.2138. HPLC purity = 94.40%, t_R_ = 6.025 min.

(*R)-3-(4-(3-methylmorpholino)-2-(1H-pyrrolo[2,3-b]pyridinedin-4-yl)-5,6,7,8-tetrahydropyrido[3,4-d]pyrimidine-7-carbonyl)cyclobutan-1-one* (**ZH-9**). White solid (90 mg). Yield 70.6%, m.p. 222–224 °C. ^1^H NMR (400 MHz, CDCl_3_) *δ* (ppm): 9.81 (s, 1H), 8.46 (d, *J* = 5.0 Hz, 1H), 8.10 (dd, *J* = 17.9, 5.0 Hz, 1H), 7.52–7.38 (m, 2H), 4.89–4.71 (m, 2H), 4.22–4.12 (m, 1H), 4.04 (d, *J* = 11.5 Hz, 1H), 3.92–3.43 (m, 10H), 3.41–3.26 (m, 2H), 2.86–2.75 (m, 2H), 1.41 (d, *J* = 6.6 Hz, 3H). ^13^C NMR (101 MHz, CDCl_3_) *δ* (ppm): 203.78, 171.56, 164.94, 161.59, 160.26, 150.18, 142.95, 137.52, 125.97, 118.42, 115.53, 114.44, 102.87, 71.08, 67.13, 51.12, 50.60, 49.96, 47.88, 43.06, 39.80, 26.18, 14.60. HRMS (ESI): *m*/*z* calcd for C_24_H_27_N_6_O_3_: 447.2145 [M + H]^+^; found: 447.2136. HPLC purity = 96.34%, t_R_ = 5.907 min.

*(R)-4-(4-(3-methylmorpholino)-2-(1H-pyrrolo[2,3-b]pyridinedin-4-yl)-5,6,7,8-tetrahydropyrido[3,4-d]pyrimidine-7-carbonyl)cyclohexan-1-one* (**ZH-10**). White solid (104 mg). Yield 76.8%, m.p. 240–242 °C. ^1^H NMR (400 MHz, CDCl_3_) (ppm): 9.53 (s, 1H), 8.45 (d, *J* = 5.3 Hz, 1H), 8.14–8.05 (m, 1H), 7.50–7.38 (m, 2H), 5.14–4.65 (m, 2H), 4.27–3.98 (m, 2H), 3.95–3.85 (m, 1H), 3.82–3.60 (m, 5H), 3.16–3.00 (m, 1H), 2.90–2.73 (m, 2H), 2.68–2.56 (m, 2H), 2.50–2.37 (m, 2H), 2.23–2.09 (m, 4H), 1.41 (d, *J* = 6.7 Hz, 3H). ^13^C NMR (101 MHz, CDCl_3_) (ppm): 209.82, 173.14, 164.94, 161.35, 160.53, 150.00, 142.83, 137.72, 125.92, 118.48, 115.57, 114.55, 102.93, 71.09, 67.13, 50.55, 50.22, 43.01, 39.97, 39.46, 38.62, 28.84, 26.20, 14.60. HRMS (ESI): *m*/*z* calcd for C_26_H_31_N_6_O_3_: 475.2458 [M + H]^+^; found: 475.2435. HPLC purity = 97.07%, t_R_ = 6.581 min.

*(R)-3-(2-(1H-indol-4-yl)-4-(3-methylmorpholino)-5,8-dihydropyrido[3,4-d]pyrimidin-7(6H)-yl)-3-oxopropanenitrile* (**ZH-12**). White solid (66 mg). Yield 55.4%, m.p. 154–156 °C. ^1^H NMR (400 MHz, DMSO-*d*_6_) *δ* (ppm): 11.25 (s, 1H), 8.14–8.06 (m, 1H), 7.53 (d, *J* = 8.1 Hz, 1H), 7.47–7.42 (m, 1H), 7.42–7.36 (m, 1H), 7.20 (t, *J* = 7.8 Hz, 1H), 4.82–4.49 (m, 2H), 4.26 (d, *J* = 5.26 Hz, 1H), 4.22 (s, 1H), 4.15–4.06 (m, 1H), 3.94–3.91 (m, 1H), 3.77–3.73 (m, 1H), 3.68–3.64 (m, 3H), 3.61–3.56 (m, 1H), 3.58–3.42 (m, 2H), 2.90–2.74 (m, 1H), 2.70 (t, *J* = 5.8 Hz, 1H), 1.26 (t, *J* = 7.7 Hz, 3H). ^13^C NMR (101 MHz, DMSO-*d*_6_) *δ* (ppm): 164.56, 162.43, 160.90, 160.66, 137.53, 129.36, 126.69, 126.58, 120.91, 120.75, 116.59, 114.17, 113.44, 103.82, 70.87, 66.88, 50.29, 49.82, 47.45, 43.14, 26.18, 25.40, 14.61. HRMS (ESI): *m*/*z* calcd for C_23_H_25_N_6_O_2_: 417.2039 [M + H]^+^; found: 417.2028. HPLC purity = 98.22%, t_R_ = 13.715 min.

#### 3.1.3. General Procedure for the Synthesis of **ZH-11** and **ZH-14**

Ethyl isocyanate (1.14 mmol) was added to a solution of **A8** (0.286 mmol) in DCM (5 mL) on an ice bath under N_2_ atmosphere. The mixture was stirred for 4 h at room temperature. After completion (monitored by TLC), H_2_O (5 mL) was added, and the mixture was extracted with DCM (3 × 5 mL). The combined organic layers were dried with Na_2_SO_4_, filtered, and concentrated under reduced pressure. The residue was purified by column chromatography on silica gel to give the compounds **ZH-11** and **ZH-14**.

*(R)-N-ethyl-4-(3-methylmorpholino)-2-(1H-pyrrolo[2,3-b]pyridin-4-yl)-5,8-dihydropyrido[3,4-d]pyrimidine-7(6H)-carboxamide* (**ZH-11**). White solid (94 mg). Yield 78.1%, m.p. 272–274 °C. ^1^H NMR (300 MHz, CDCl_3_) *δ* (ppm): 10.33 (s, 1H), 8.44 (d, *J* = 5.1 Hz, 1H), 8.07 (d, *J* = 5.0 Hz, 1H), 7.46 (d, *J* = 3.5 Hz, 1H), 7.40 (d, *J* = 3.5 Hz, 1H), 4.74–4.51 (m, 3H), 4.13 (d, *J* = 6.3 Hz, 1H), 4.07–3.98 (m, 1H), 3.99–3.90 (m, 1H), 3.90–3.83 (m, 1H), 3.82–3.69 (m, 2H), 3.69–3.64 (m, 1H), 3.54–3.42 (m, 1H), 3.42–3.31 (m, 2H), 2.76 (t, *J* = 5.5 Hz, 2H), 2.30 (s,1H), 1.37 (d, *J* = 6.7 Hz, 3H), 1.21 (t, *J* = 7.2 Hz, 3H). ^13^C NMR (101 MHz, DMSO-*d*_6_) *δ* (ppm): 164.66, 162.33, 160.49, 157.40, 150.66, 142.73, 136.86, 127.55, 118.15, 115.18, 114.81, 102.41, 70.85, 66.84, 50.19, 49.00, 42.95, 35.43, 26.51, 16.07, 14.61. HRMS (ESI): *m*/*z* calcd for C_22_H_28_N_7_O_2_: 422.2304 [M + H]^+^; found: 422.2294. HPLC purity = 98.52%, t_R_ = 12.007 min.

*(R)-N-ethyl-2-(1H-indol-4-yl)-4-(3-methylmorpholino)-5,8-dihydropyrido[3,4-d]pyrimidine-7(6H)-carboxamide* (**ZH-14**). White solid (106 mg). Yield 88.0%, m.p. 200–202 °C. ^1^H NMR (400 MHz, CDCl_3_) *δ* (ppm): 8.54 (s, 1H), 8.19 (d, *J* = 7.2 Hz, 1H), 7.56–7.49 (m, 2H), 7.40–7.28 (m, 2H), 4.70–4.50 (m, 2H), 4.15–4.06 (m, 1H), 4.05–3.91 (m, 2H), 3.89–3.86 (m, 1H), 3.81–3.75 (m, 1H), 3.69–3.56 (m, 2H), 3.51–3.42 (m, 1H), 3.42–3.33 (m, 2H), 2.74 (t, *J* = 5.5 Hz, 2H), 1.91 (s, 1H), 1.36 (d, *J* = 6.7 Hz, 3H), 1.22 (t, *J* = 7.2 Hz, 3H). ^13^C NMR (101 MHz, CDCl_3_) *δ* (ppm): 164.85, 162.81, 160.55, 157.31, 137.03, 130.00, 126.51, 125.03, 121.69, 121.46, 113.65, 113.27, 104.38, 71.18, 67.22, 50.50, 49.02, 43.20, 40.20, 35.87, 26.39, 15.60, 14.45. HRMS (ESI): *m*/*z* calcd for C_23_H_29_N_6_O_2_: 421.2352 [M + H]^+^; found: 421.2337. HPLC purity = 95.46%, t_R_ = 14.789 min.

### 3.2. Biological Activity Assay

#### 3.2.1. Molecular Modeling

Molecular docking was conducted by the docking module of the Schrodinger Maestro 2018 in the X-ray crystal structure of the PI3Kα mutant mimicking ATR (PDB: 5UK8), which was retrieved from the Protein Data Bank. The Protein preparation wizard module prepared the target protein structure with polar hydrogen added and water removed from the protein. The energy was minimized and optimized using the OPLS3 force field. The receptor grid was generated using Receptor Grid Generation. The Ligand Preparation Wizard carried out the three-dimensional structures of compounds of interest for molecular docking. The types of interactions between the compounds of interest with protein were analyzed at the end of molecular docking.

#### 3.2.2. Cell Lines and Culture Methods

A549, HCT116, NCI-H23, and LoVo cells were purchased from the Cell Resources Center of Shanghai Academy of Life Sciences, Shanghai, China. A549 cells were cultured in McCoy’s5A medium supplemented with 10% FBS (KeyGEN Biotech, Nanjing, China) and 1% penicillin/streptomycin (Gibco, 15070-063). HCT116 cells were cultured in IMDM medium supplemented with 10% FBS (KeyGEN Biotech, Nanjing, China) and 1% penicillin/streptomycin (Gibco, 15070-063). NCI-H23 cells were cultured in RPMI 1640 medium supplemented with 10% FBS (KeyGEN Biotech, Nanjing, China) and 1% penicillin/streptomycin (Gibco, 15070-063). LoVo cells were cultured in F12K medium supplemented with 10% FBS (KeyGEN Biotech, Nanjing, China) and 1% penicillin/streptomycin (Gibco, 15070-063). All cells were cultured in an incubator at 37 °C under a 5% CO_2_ atmosphere.

#### 3.2.3. Cell Proliferation Assay

For the assay, 3000/well cells were planted into 96-well plates for 24 h and then treated with the indicated doses of drugs or controls for 72 h. Following the manufacturer’s instructions, cell proliferation/survival was measured using the CCK-8 (KeyGEN Biotech, Nanjing, China) assay and plotted via GraphPad 8.0.

#### 3.2.4. Colony Formation Assay

LoVo cells at a concentration of 800 cells/well were exposed to various concentrations of **ZH-12** for 14 days. After 14 days of treatment, the colonies were fixed with 4% paraformaldehyde, stained with 0.1% crystal violet, and imaged using an upright biological microscope (Olympus BX53, Tokyo, Japan). The experiments were repeated at least three times and plotted via GraphPad 8.0.

#### 3.2.5. Migration Assays

LoVo cells in the logarithmic phase were collected and seeded in 6-well plates and cultured overnight. A straight line was drawn across the cell with a sterile pipette tip. A culture medium containing different concentrations of **ZH-12** or 0.1% DMSO (10% FBS) was added. Scratch widths were recorded under an inverted microscope (Olympus, Waltham, MA, USA) at 0 h and 48 h.

#### 3.2.6. Comet Assay

The comet assay was performed following the protocol of the manufacturer of the Comet Assay Kit (KeyGEN Biotech, Nanjing, China). LoVo cells were treated with AZD6738 and different concentrations of **ZH-12** for 3 days. After 3 days of treatment, the cells (1 × 10^4^/mL) were collected, mixed with low-melting agarose at a ratio of 1:10 (*v*/*v*), layered on slides, lysed in lysis buffer at 4 °C for 2 h, and then lysed in alkaline lysis solution at room temperature for 30 min. Following electrophoresis, the cells were stained with PI and observed with an inverted biomicroscope (Olympus BX53, Tokyo, Japan). Three images were randomly captured per slide.

#### 3.2.7. Cell Apoptosis

The percentage of apoptotic cells was determined by the Annexin V-FITC/PI double staining kit (KeyGEN Biotech, Nanjing, China). According to the manufacturer’s instructions, LoVo cells were stained with Annexin V-FITC and PI. The signal of the FITC and PI was recorded by BD FACSCelesta Flow Cytometer (BD FACSCelesta, Becton, Dickinson and Company) and the FlowJo software.

#### 3.2.8. Western Blotting

LoVo cells with a density of 1 × 10^6^ were inoculated in a small cell culture dish at a single density for 24 h and then treated with the specified concentration of 5% dimethyl sulfoxide or compound at different times. The cells were washed with cold PBS and lysed with RIPA buffer (Beyotime, Shanghai, China) containing protease inhibitors (Beyotime, Shanghai, China) and phosphatase inhibitors (Beyotime, Shanghai, China). After lysis of the total cell solution on ice for 60 min, the lysate was centrifuged (12,000 rpm, 4 °C, 20 min); The total protein concentrations obtained were determined using the BCA kit (Beyotime, Shanghai, China). A proper 6 × loading buffer was added before use, and the samples were denatured by boiling. Then, 30–100 μg of protein was loaded onto a 4–12% SDS-PAGE gel and then transferred to the PVDF membrane (Millipore). The membrane was occluded for 1 h, incubated overnight with primary antibody at 4 °C, followed by washing 3 times with Tris-buffered saline and Tween 20 (TBST) for 10 min. After incubation with a second antibody with horseradish peroxidase for 60 min at room temperature, the membrane was washed 3 times for 10 min with TBST. Finally, blotting was performed with an enhanced chemiluminescence assay kit (KeyGEN Biotech, Nanjing, China). The software ImageJ was used to quantify the percentage degradation of proteins. The primary antibodies used were ATR (1/1000 dilution, CST, #2790), Phospho-ATR (Thr1989) (1/1000 dilution, CST, #30632), CHK1 (1/1000 dilution, CST, #2360), Phospho-CHK1 (Ser345) (1/1000 dilution, CST, #2348), and β-tubulin (1/5000 dilution, CST, #5536).

#### 3.2.9. Efficacy and Pharmacodynamics Studies in the LoVo Xenograft Model in Mice

Balb/c nude mice (18–22 g, female, 4 weeks old) were purchased from Hangzhou Medical College (Hangzhou, China). All mice were inoculated subcutaneously in the right flank with 5 × 10^6^ LoVo cells to initiate tumor growth. When the mean tumor volume reached approximately 100 mm^3^, all mice were randomly divided into 8 groups (*n* = 5 per group) based on body weight and volume. AZD6738, Cisplatin, and **ZH-12** were dissolved in 10% DMSO, 5% Tween 80, 30% PEG300, and 55% saline as formulations. AZD6738 and **ZH-12** were administered twice daily for 18 consecutive days at the indicated doses. Cisplatin was administered twice a week at the indicated doses. Tumor size and animal weight were measured every 3 days. Tumor volume was calculated as tumor volume (cm^3^) = (length × width^2^)/2. Tumor inhibition rate (%) = (1 − mean tumor weight in the treatment group/mean tumor weight in the control group) × 100%. The results were obtained from 5 test mice (mean ± SD) and calculated using GraphPad 8.0 software.

#### 3.2.10. H&E Staining

Tumor samples from LoVo xenograft model mice were collected on day 18 in the balb/c mouse model. Tumor samples were fixed in 4% paraformaldehyde for 48 h, dehydrated in ethanol, immersed in xylene, embedded in paraffin, and sectioned longitudinally. The paraffin-embedded sections were stained for H&E according to the manufacturer’s instructions (Beyotime, Shanghai, China). Samples were photographed using a DM6B positive fluorescence microscope (Leica, Wetzlar, Germany). Three images were randomly captured per slide.

#### 3.2.11. Immunohistochemical Analysis

Tumors from LoVo xenograft model mice were embedded in paraffin and cut into longitudinal sections. The paraffin-embedded sections were incubated with 0.3% hydrogen peroxide for 30 min to block endogenous peroxidase and then 1.0% BSA to block it. Following blocking, the paraffin-embedded sections were incubated overnight at 4 °C with the primary antibody (anti-γH2AX antibody), and the secondary antibody was incubated for an additional 60 min at room temperature and then counterstained for 60 s with hematoxylin. Each group was examined using a DM6B positive fluorescence microscope (Leica, Frankfurt, Germany). Five images were randomly collected per slide. The percentage of stained spots was analyzed by Image J V1.8.0.112 software.

## 4. Conclusions

In conclusion, a series of novel ATR inhibitors containing a 5,6,7,8-tetrahydropyrido[4,3-*d*]pyrimidin-4-yl scaffold was designed, synthesized, and evaluated for their biological activities in vitro and in vivo. Among them, **ZH-12** exhibited the most potent ATR inhibitory activity with an IC_50_ value of 0.0068 μM and high selectivity for ATR against 207 other protein kinases. In the in vitro assay, **ZH-12** showed more potent antiproliferative activity in ATM-deficient tumor cells than in ATM-normal tumor cells. Furthermore, in the in vivo assay, **ZH-12** showed good antitumor activity as a single agent or in combination with cisplatin in xenograft LoVo tumor models. Overall, **ZH-12** can be a promising lead compound that deserves further in-depth study.

## Figures and Tables

**Table 1:**
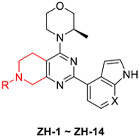
The in vitro inhibitory activities of compounds **ZH-1**~**ZH-14** against ATR.

Compounds	X	R	ATR IC_50_ ± SD (nM) ^a^	clogP ^b^
**ZH-1**	N	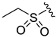	53.26 ± 1.08	2.27
**ZH-2**	N	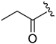	41.60 ± 1.38	2.80
**ZH-3**	N	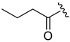	42.49 ± 1.05	3.22
**ZH-4**	N	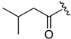	7.31 ± 0.16	3.55
**ZH-5**	N	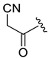	10.81 ± 0.72	2.35
**ZH-6**	N	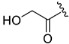	34.03 ± 0.27	1.45
**ZH-7**	N	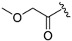	9.23 ± 0.16	1.81
**ZH-8**	N	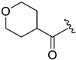	27.47 ± 1.04	2.44
**ZH-9**	N	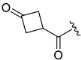	16.04 ± 1.08	2.21
**ZH-10**	N	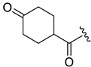	11.92 ± 0.19	3.05
**ZH-11**	N	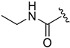	11.42 ± 0.25	2.39
**ZH-12**	CH	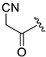	6.81 ± 0.04	2.94
**ZH-13**	CH	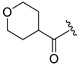	214.66 ± 0.04	2.49
**ZH-14**	CH	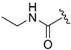	14.86 ± 0.29	2.99
**AZD6738**	/	/	6.50 ± 0.28	

^a^ IC_50_ values are expressed as the mean ± SD of two independent experiments. ^b^ clogP was calculated by ChemDraw 2020.

**Table 2:** Inhibitory effects of selected compounds against A549, HCT116, NCI-H23, and LoVo cell lines.

Compound	IC_50_ ± SD (μM) ^a^			
	A549	HCT116	NCI-H23	LoVo
**ZH-4**	20.85 ± 1.21	5.17 ± 0.58	1.74 ± 0.05	18.00 ± 1.69
**ZH-7**	25.63 ± 1.62	6.02 ± 0.26	6.77 ± 0.43	5.71 ±0.81
**ZH-12**	4.74 ± 0.27	1.61 ± 0.08	0.77 ± 0.13	0.29 ± 0.01
**AZD6738**	6.60 ± 0.24	4.32 ± 0.16	0.50 ± 0.06	0.41 ± 0.01

^a^ IC_50_ values are expressed as the mean ± SD of three independent experiments.

## Data Availability

Not applicable.

## Supplementary Materials

The following supporting information can be downloaded at: https://www.mdpi.com/article/10.3390/molecules28114521/s1, the synthesis conditions of intermediates **A2**~**A8** and the spectra for the target compounds.

## Sample Availability

Samples of all compounds are available from the authors.
