# Supplementary material for: Design, Synthesis, and Biological Evaluation of Potent and Selective Inhibitors of Ataxia Telangiectasia Mutated and Rad3-Related (ATR) Kinase for the Efficient Treatment of Cancer
Source: Molecules. 2023 Jun 2;28(11):4521. doi: 10.3390/molecules28114521 (PMC10254649; doi:10.3390/molecules28114521)

Supplemental files for

Design, synthesis, and biological evaluation of potent and selective inhibitors of ataxia telangiectasia mutated and Rad3-Related (ATR) kinase for efficient treatment of cancer

Jialu Shao[1,†], Lei Huang[1,2,†], Wenwen Lai[1], Yi Zou[1,*], Qihua Zhu[1,3,*]

1 Department of Medicinal Chemistry, School of Pharmacy, China Pharmaceutical University, Nanjing 210009, China; shaojialu1997@126.com (J.S.); huangleiphd2019@163.com (L.H.); laiwenw@126.com (W.L.)

2 Department of Pharmacology and Medicinal Chemistry, Jiangsu Vocational College of Medicine, Yancheng 224005, China

3 Jiangsu Key Laboratory of Drug Design and Optimization, Department of Medicinal Chemistry, China Pharmaceutical University, Nanjing 211198, China

* Correspondence: zouyi@cpu.edu.cn (Y.Z.); zhuqihua@vip.126.com (Q.Z.)

† These authors contributed equally to this work.

Contents

1. Synthesis of All Intermediates……………………………………………………2

2. ^{1}H NMR, ^{13}C NMR and HRMS Spectra of All Target Compounds…...……....…4

1. Synthesis of All Intermediates

General method of chemistry

All reagents and solvents were purchased from commercial suppliers and used without further purification. All reaction progress was monitored by TLC on silica gel plates (silica gel 60 F_{254}) with UV (254 or 365 nm) or other suitable stain visualization. ^{1}H NMR (300 or 400 MHz) and ^{13}C NMR (75 MHz or 100 MHz) spectra were recorded in $CDCl_3$ or DMSO-d_6 with TMS as internal standard. Chemical shifts are reported in δ scale (ppm) relative to internal TMS, Coupling constants (J) values are given in Hertz, and spin multiplicities are expressed as s (singlet), d (doublet), t (triplet), q (quartet), dd(doublet of doublet), dt(doublet of triplet) or m (multiplet). Low and high-resolution mass spectra were recorded by ESI-MS.

*7-benzylhexahydropyrido[3,4-d]pyrimidine-2,4(1H,3H)-dione (**A2**)*

To a solution of **A1** (15.0 g, 50.4 mmol) and urea (7.26 g, 119.8 mmol) in MeOH (150 mL) in a three-necked flask, 5.4 mol/L NaOMe in MeOH (22.3 mL, 119.8 mmol) was dropped at 0 ^oC under N_2 atmosphere. Then the mixture warmed to room temperature, and stirred at reflux for 8 h. After completion, the mixture cooled to room temperature and stirred for another 1 h, then filtered under reduced pressure. The filter cake was resolved in H_2O (100 mL), adjusted the pH to neutral with 2 mol/L HCl, and filtered. The filter cake was dried to give compound **A2** as white solid, 10.8 g, 83.1% yield, used for the next step without purification. MS (ESI): m/z 260.1 $[M+H]^+$; found: 260.2.

*7-benzyl-2,4-dichloro-5,6,7,8-tetrahydropyrido[3,4-d]pyrimidine (**A3**)*

A2 (7.7 g, 29.9 mmol) was added to $POCl_3$ (60 mL) in batches at 0 ^oC. Then the mixture warmed to room temperature, and stirred at reflux for 12 h. After completion, the mixture cooled to room temperature, and concentrated under reduced pressure to remove $POCl_3$. The residue was poured to ice. The mixture was adjusted to ~8 by saturated aqueous $NaHCO_3$. The aqueous layer was extracted with EA (3 × 100 mL). The combined organic layers were washed with brine (100 mL), dried by Na_2SO_4, filtered, and concentrated under reduced pressure to give compound ATR-2a-3a as brown liquid, 6.3 g, 71.5% yield, used in the next step without purification. MS (ESI): m/z 294.0 $[M+H]^+$; found: 294.0.

*(R)-4-(7-benzyl-2-chloro-5,6,7,8-tetrahydropyrido[3,4-d]pyrimidin-4-yl)-3-methylmorpholine(**A5**)*

To a solution of **A3** (12.0 g, 48.0 mmol) in MeCN (90 mL) in a 350 mL seal, Na_2CO_3 (12.8 g, 120.4 mmol) and (R)-3-methylmorpholine (**A4**) (5.85 g, 57.8 mmol) was added. Then the mixture stirred overnight under room temperature. After completion, 100 mL H_2O was add to the mixture, and concentrated under reduced pressure to remove MeCN. Then, the residue was extracted with DCM (3 × 100 mL). The combined organic layers were dried with Na_2SO_4, filtered, and concentrated under reduced pressure. The residue was purified by column chromatography on silica gel to give compound **A5** as white solid, 9.5 g, 64.9% yield, m.p. 112 - 114 ℃. ^{1}H NMR (300 MHz, $CDCl_3$) δ 7.41 – 7.30 (m, 5H), 4.13 (q, J = 7.3 Hz, 1H), 3.92 (dd, J = 12.1, 3.9 Hz, 1H), 3.75 (d, J = 2.9 Hz, 1H), 3.72 – 3.70 (m, 2H), 3.68 (d, J = 4.8 Hz, 2H), 3.65 (d, J = 2.4 Hz, 1H), 3.61 (d, J = 2.4 Hz, 1H), 3.58 (d, J = 2.3 Hz, 1H), 3.49 (dd, J = 13.6, 2.3 Hz, 1H), 2.80 – 2.61 (m, 4H), 1.32 (d, J = 6.8 Hz, 3H). MS (ESI): m/z 259.2 $[M+H]^+$; found: 259.0.

*(R)-4-(7-benzyl-2-(1H-indol-4-yl)-5,6,7,8-tetrahydropyrido[3,4-d]pyrimidin-4-yl)-3-methylmorpholine (**A7-1**)*

To a solution of **A5** (0.46 g, 1.29 mmol) in EtOH (10 mL), indole-4-boronic acid pinacol ester (**A6-1**) (0.36 g, 1.48 mmol), $Pd(PPh_3)_2Cl_2$ (90 mg, 0.129 mmol) and Na_2CO_3 (0.34 g, 3.22 mmol, dissolved in 2 mL H_2O) were added. The mixture was stirred at reflux under N_2 for 12 h. After completion, the mixture diluted by water to dissolve Na_2CO_3, and then was filtered. The filter cake was slurry with EtOH to give **A7-1** as white solid, 0.43 g, 76.3% yield, m.p. 224 - 226 ℃. ^{1}H NMR (400 MHz, $CDCl_3$) δ (ppm): 8.31 (s, 1H), 8.12 (d, J = 7.6 Hz, 1H), 7.67 (dd, J = 12.0, 6.9 Hz, 1H), 7.54 (q, J = 6.6 Hz, 1H), 7.50 – 7.44 (m, 3H), 7.39 – 7.33 (m, 3H), 7.29 (d, J = 4.5 Hz, 1H), 4.11 (s, 1H), 3.96 (d, J = 11.0 Hz, 1H), 3.90 – 3.82 (m, 2H), 3.80 – 3.73 (m, 2H), 3.71 – 3.66 (m, 2H), 3.61 (s, 1H), 3.58 (d, J = 7.0 Hz, 1H), 3.54 – 3.46 (m, 1H), 3.01 – 2.56 (m, 4H), 1.32 (d, J = 6.8 Hz, 3H). ^{13}C NMR (101 MHz, DMSO-d_6) δ (ppm): 164.49, 162.96, 162.08, 138.57, 137.47, 129.72, 129.31, 128.77, 127.56, 126.66, 126.43, 120.87, 120.66, 113.90, 113.69, 103.76, 70.95, 66.94, 61.86, 58.49, 49.99, 43.19, 26.75, 14.51. MS (ESI): m/z 440.2 [M + H]$^+$; found: 440.2.

*(R)-4-(2-(1H-indol-4-yl)-5,6,7,8-tetrahydropyrido[3,4-d]pyrimidin-4-yl)-3-methylmorpholine (**A8-1**)*

To a solution of **A7-1** (0.28 g, 0.67 mmol) in 5 mL HCOOH/H_2O (1:5), 10% Pd/C (0.1g, 0.094 mmol) was added. The mixture was stirred at 30 ^{o}C under H_2 atomosphere for 6 h. After completion, the mixture was filtered through Celite, washed with DCM/MeOH (5/1) (30 mL), and the filtrate was concentrated under reduced pressure. The residue was purified on a silica gel column to provide **A8-1** as white solid, 0.18 g, 80.8 % yield, m.p. 146 - 148 ℃. ^{1}H NMR (300 MHz, $CDCl_3$) δ (ppm): 8.40 (s, 1H), 8.16 (dd, J = 7.5, 1.0 Hz, 1H), 7.51 (d, J = 3.0 Hz, 1H), 7.47 (d, J = 8.1 Hz, 1H), 7.32 – 7.27 (m, 2H), 4.19 (d, J = 4.9 Hz, 2H), 4.14 – 4.03 (m, 1H), 3.97 (dt, J = 11.4, 3.0 Hz, 1H), 3.87 (dd, J = 11.1, 3.2 Hz, 1H), 3.82 – 3.73 (m, 1H), 3.68 (dd, J = 11.2, 3.0 Hz, 1H), 3.61 – 3.53 (m, 2H), 3.23 (dt, J = 12.5, 5.0 Hz, 1H), 3.03 (dt, J = 12.3, 6.1 Hz, 1H), 2.71 (t, J = 5.6 Hz, 1H), 2.63 (s, 1H), 1.31 (d, J = 6.6 Hz, 3H). ^{13}C NMR (101 MHz, $CDCl_3$) δ (ppm): 164.98, 162.69, 161.92, 136.98, 130.29, 126.54, 124.89, 121.74, 121.48, 113.84, 113.05, 104.56, 71.31, 67.29, 50.40, 49.96, 43.59, 43.19, 26.34, 14.46. HRMS (ESI): m/z 350.2 [M + H]$^+$; found: 350.2.

(R)-4-(7-benzyl-2-(1H-pyrrolo[2,3-b]pyridin-4-yl)-5,6,7,8-tetrahydropyrido[3,4-d]pyrimidin-4-yl) -3-methylmorpholine (A7-2)

Intermediate **A7-2** was prepared similarly as described for intermediate **A7-1**. White solid, 1.00 g, 81.5% yield, m.p. >250 ℃. ^{1}H NMR (300 MHz, $CDCl_3$) δ 9.64 (s, 1H), 8.42 (d, J = 5.0 Hz, 1H), 8.04 (d, J = 5.0 Hz, 1H), 7.49 – 7.30 (m, 7H), 4.21 – 4.06 (m, 1H), 4.04 – 3.90 (m, 2H), 3.90 – 3.83 (m, 1H), 3.80 (s, 2H), 3.76 (s, 1H), 3.75 – 3.67 (m, 2H), 3.67 – 3.57 (m, 2H), 2.96 – 2.86 (m, 1H), 2.85 – 2.77 (m, 2H), 2.69 – 2.57 (m, 1H), 1.34 (d, J = 6.7 Hz, 3H). MS (ESI): m/z 441.2 [M+H]$^+$; found: 441.3.

*(R)-4-(2-(1H-pyrrolo[2,3-b]pyridin-4-yl)-5,6,7,8-tetrahydropyrido[3,4-d]pyrimidin-4-yl)-3-methyl morpholine (**A8-2**)*

Intermediate **A8-2** was prepared similarly as described for intermediate **A7-1**. White solid, 0.18 g, 75.4 % yield, m.p. 170 - 173 ^{o}C. ^{1}H NMR (300 MHz, $CDCl_3$) δ 9.55 (s, 1H), 8.41 (d, J = 5.0 Hz, 1H), 8.03 (d, J = 5.0 Hz, 1H), 7.47 – 7.29 (m, 2H), 4.18 – 4.08 (m, 1H), 4.03 – 3.93 (m, 1H), 3.92 – 3.86 (m, 1H), 3.86 – 3.79 (m, 1H), 3.76 (s, 2H), 3.73 (s, 2H), 3.67 (s, 1H), 3.66 – 3.54 (m, 2H), 2.80 – 2.68 (m, 1H), 2.67 – 2.54 (m, 1H), 1.33 (d, J = 6.7 Hz, 3H). MS (ESI): m/z 351.2 [M+H]$^+$; found: 351.1.

2. ^{1}H NMR, ^{13}C NMR and HRMS Spectra of All Target Compounds

^{1}H-NMR of compound **ZH-1**

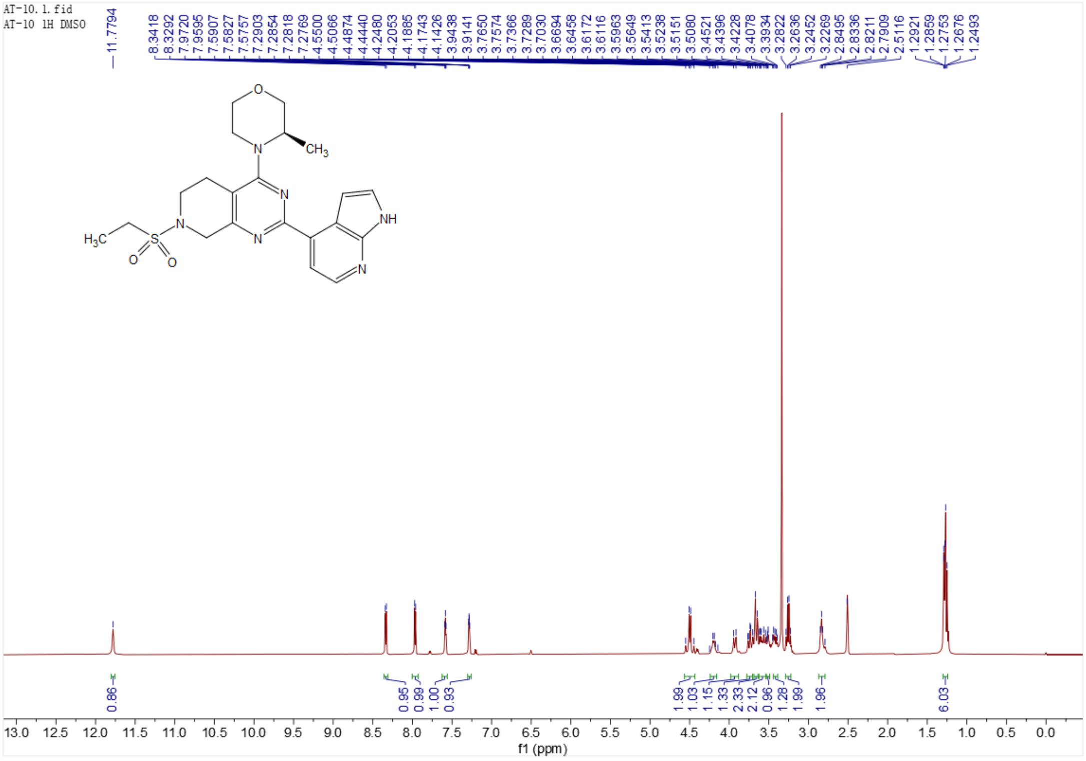

^{13}C-NMR of compound **ZH-1**

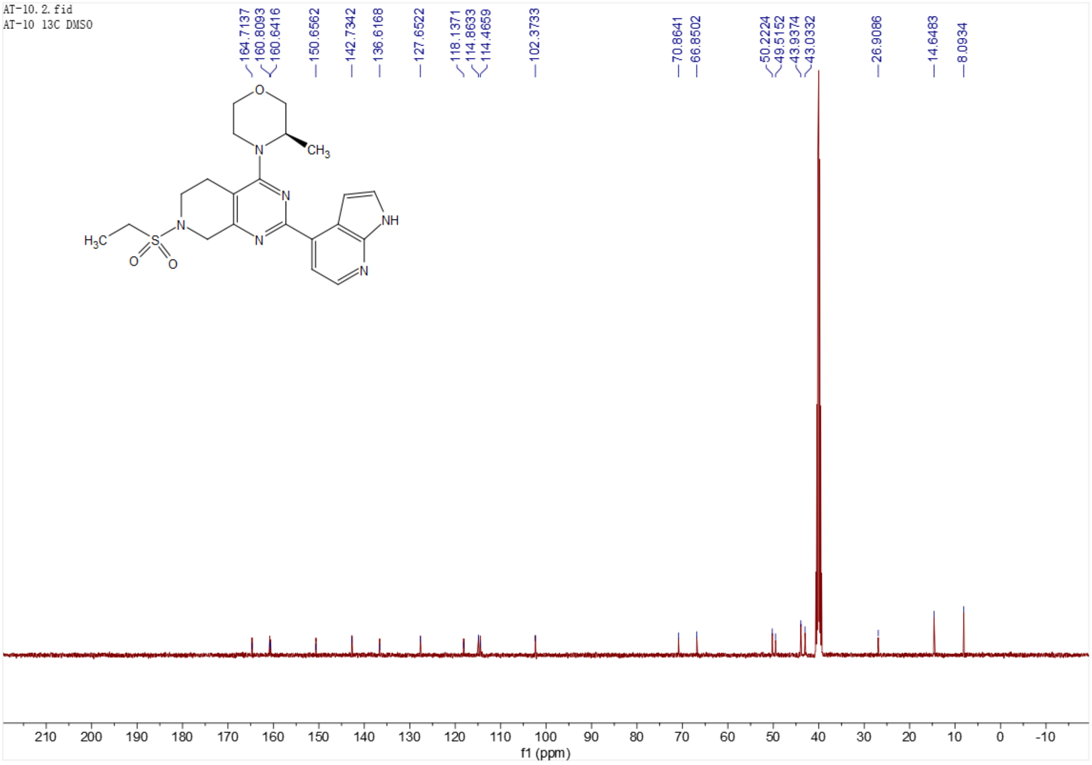

HRMS of compound **ZH-1**

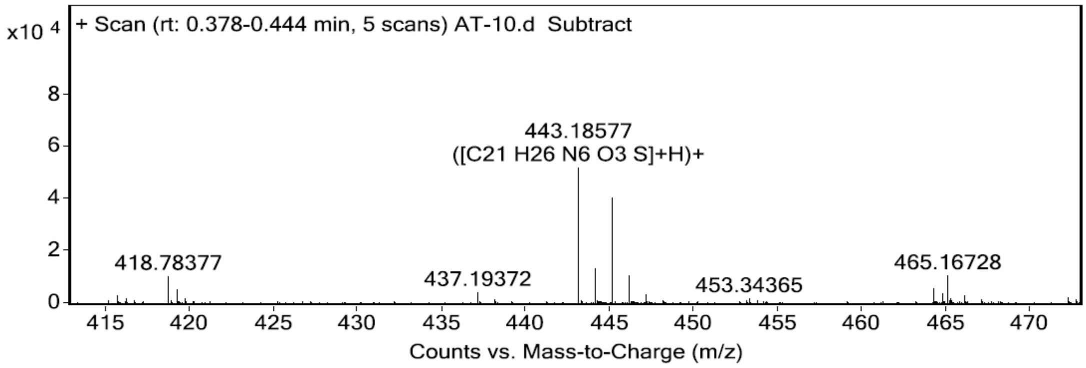

^{1}H-NMR of compound **ZH-2**

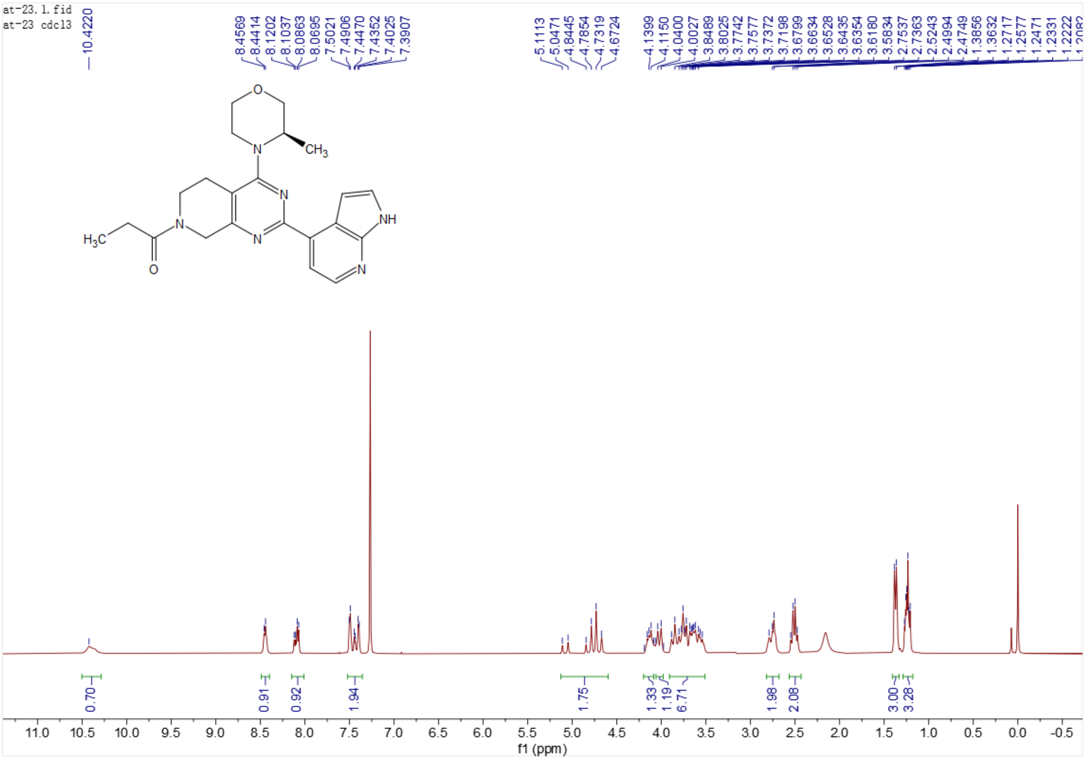

^{13}C-NMR of compound **ZH-2**

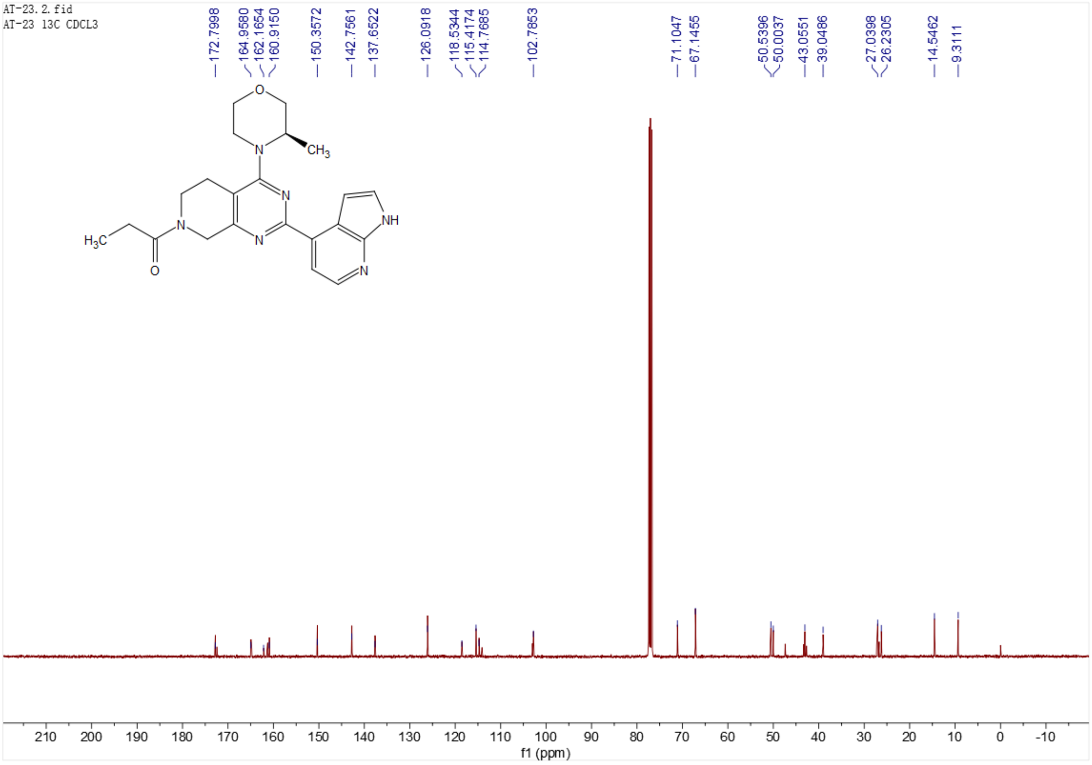

HRMS of compound **ZH-2**

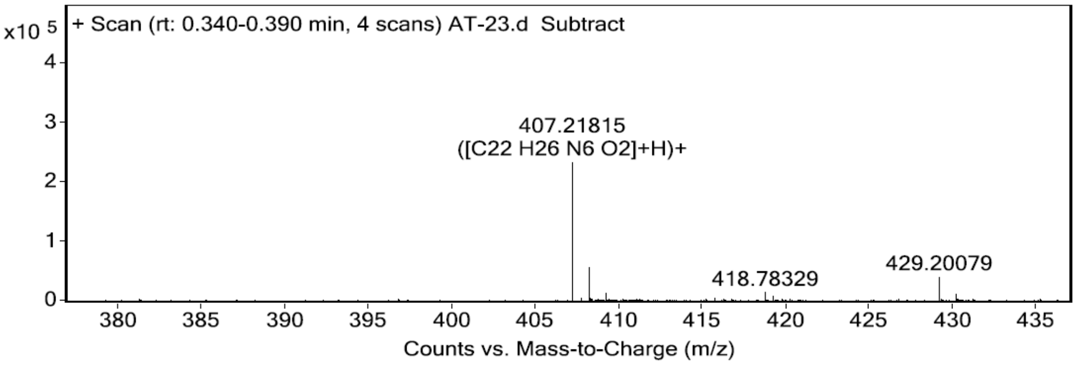

^{1}H-NMR of compound **ZH-3**

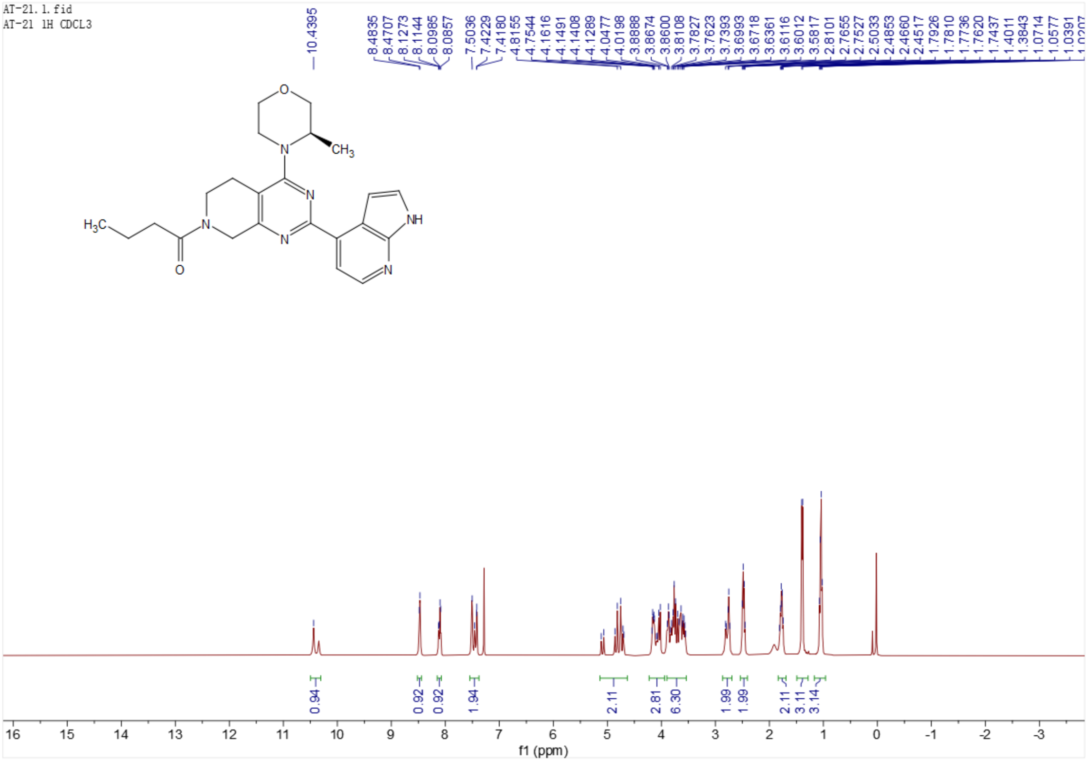

^{13}C-NMR of compound **ZH-3**

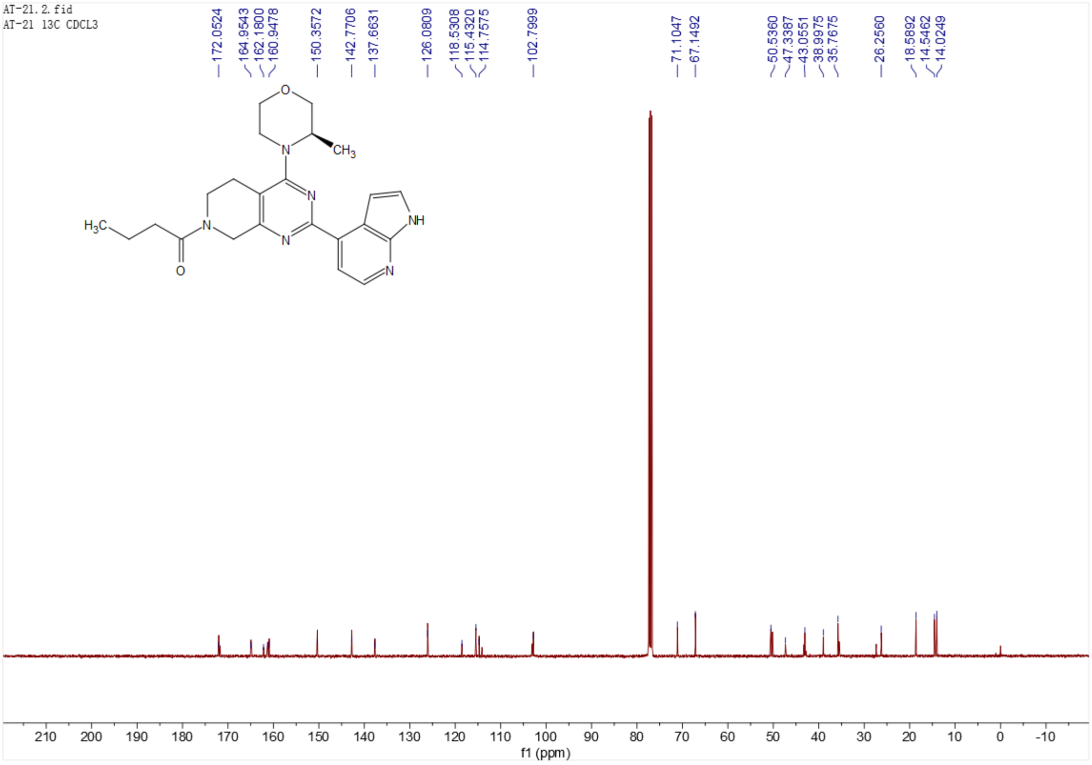

HRMS of compound **ZH-3**

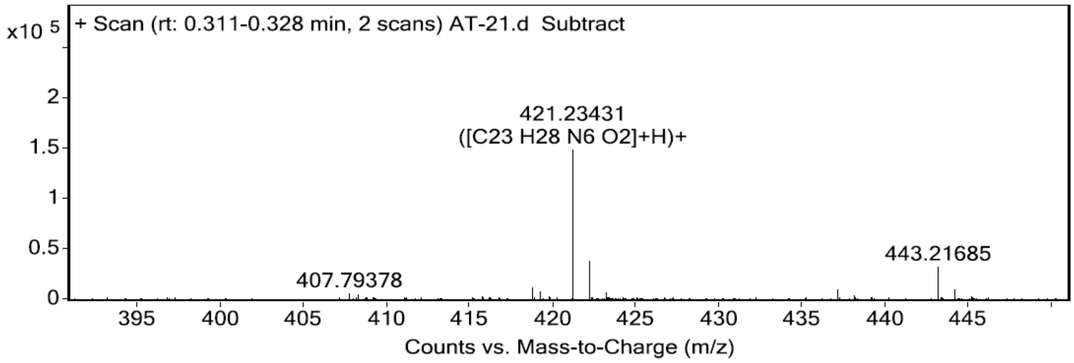

^{1}H-NMR of compound **ZH-4**

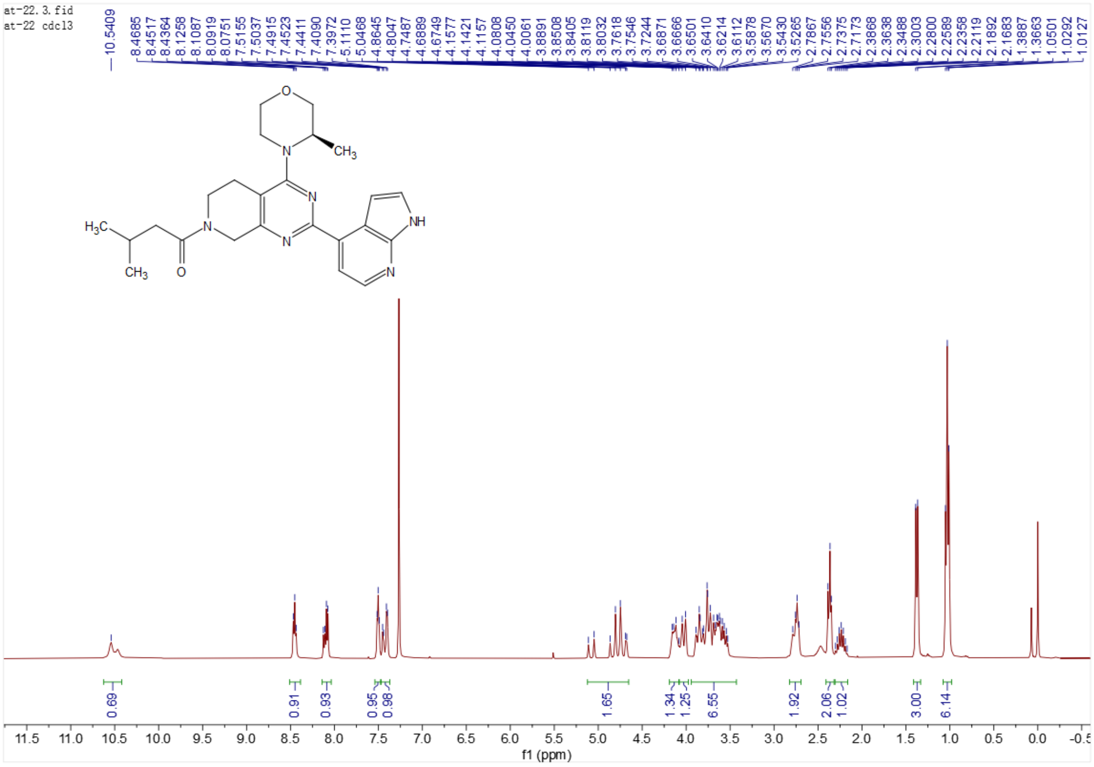

^{13}C-NMR of compound **ZH-4**

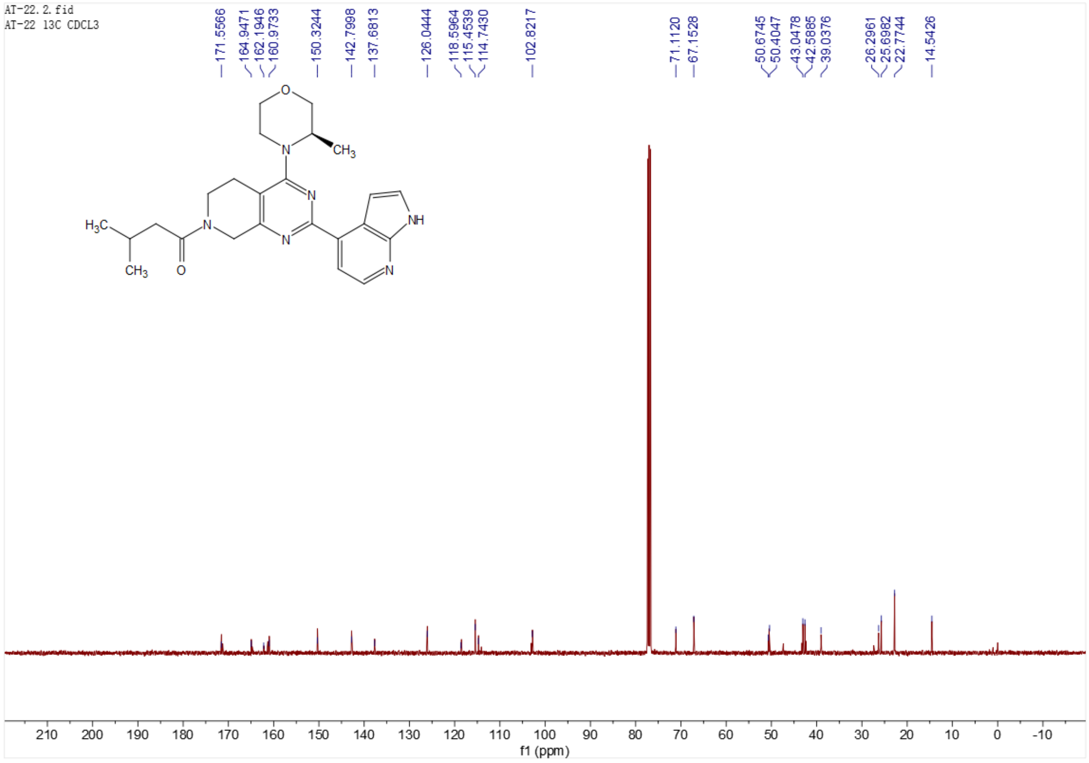

HRMS of compound **ZH-4**

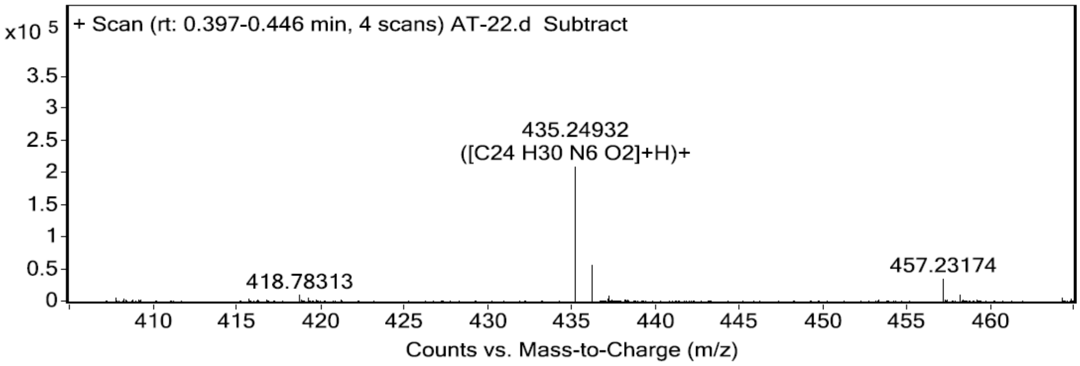

^{1}H-NMR of compound **ZH-5**

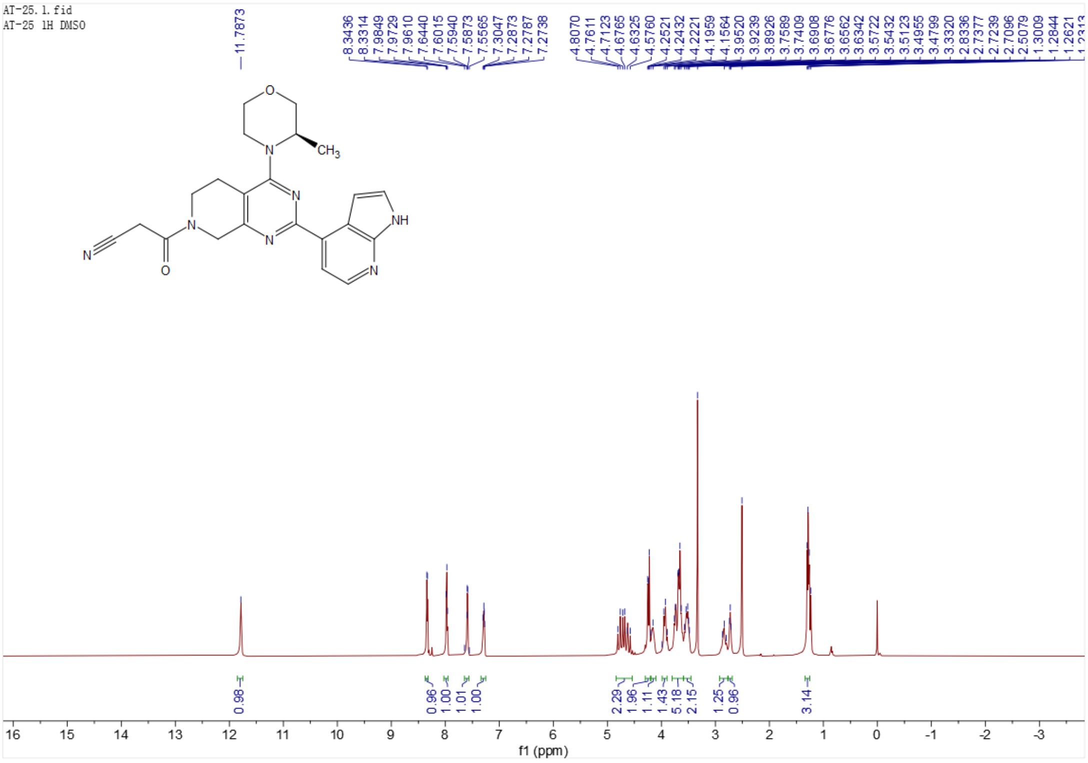

^{13}C-NMR of compound **ZH-5**

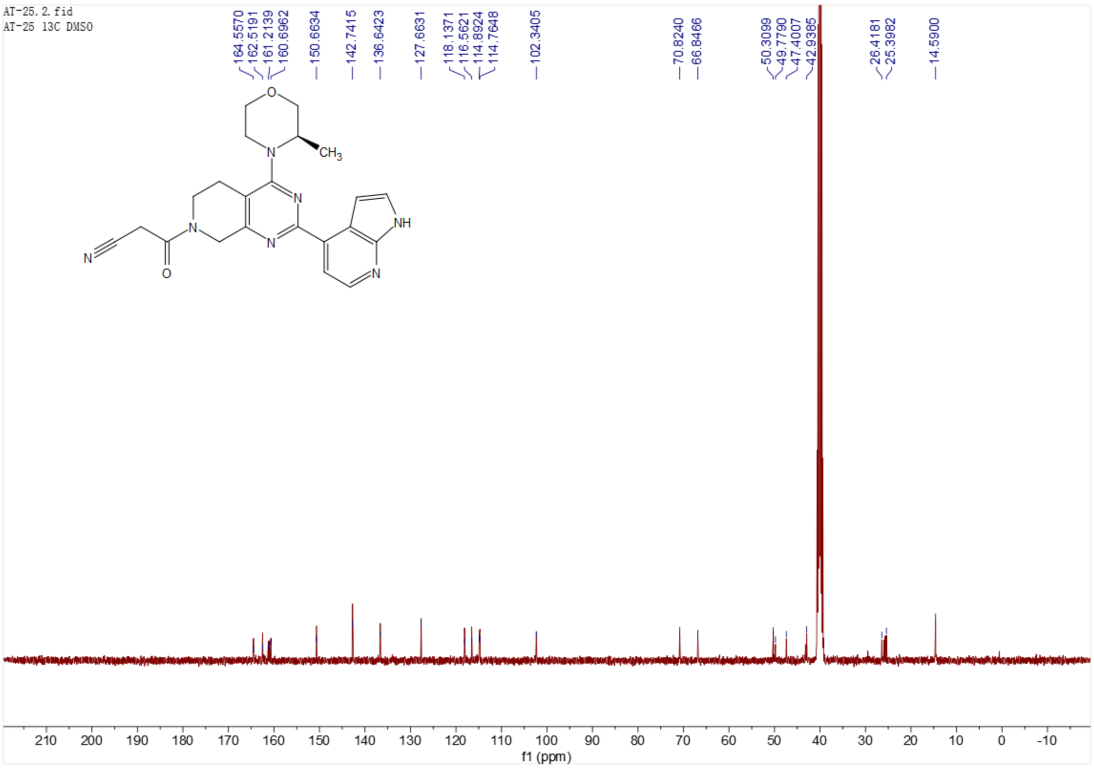

HRMS of compound **ZH-5**

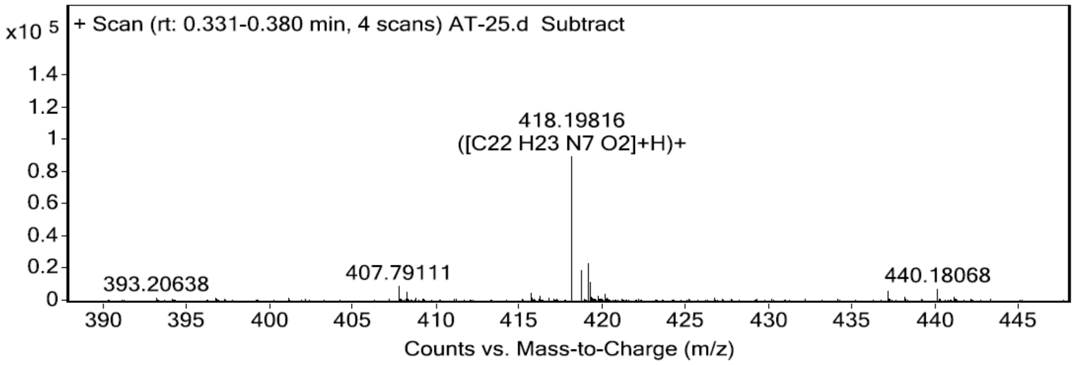

^{1}H-NMR of compound **ZH-6**

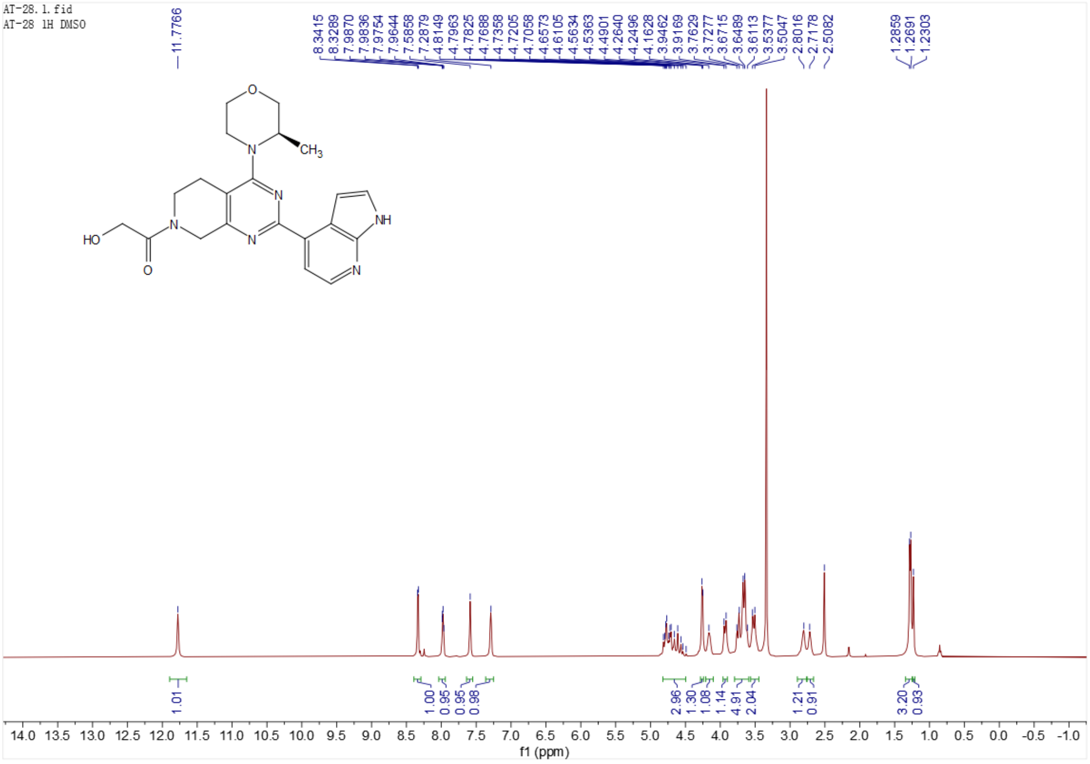

^{13}C-NMR of compound **ZH-6**

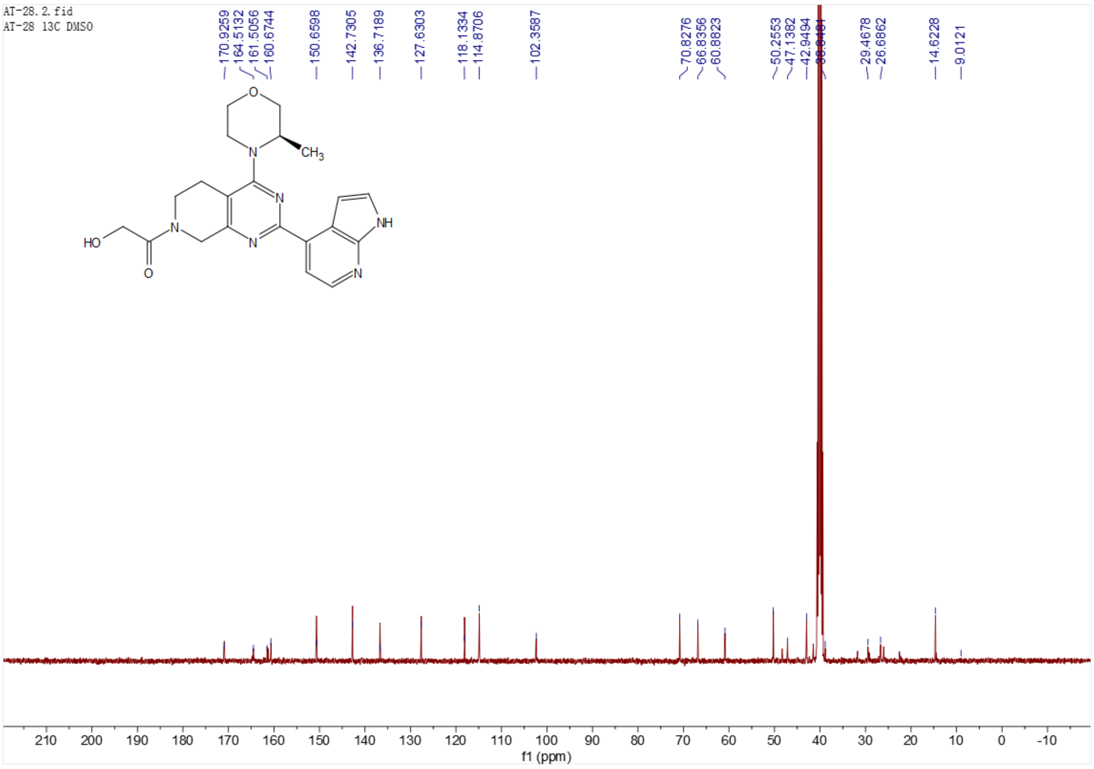

HRMS of compound **ZH-6**

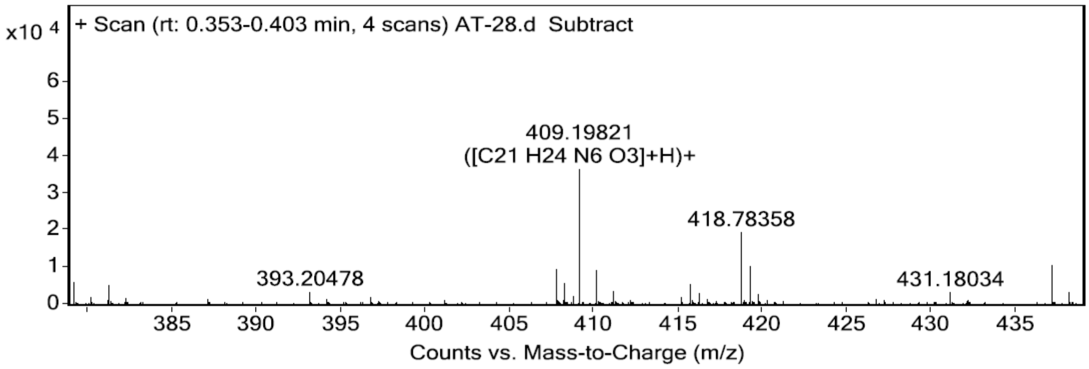

^{1}H-NMR of compound **ZH-7**

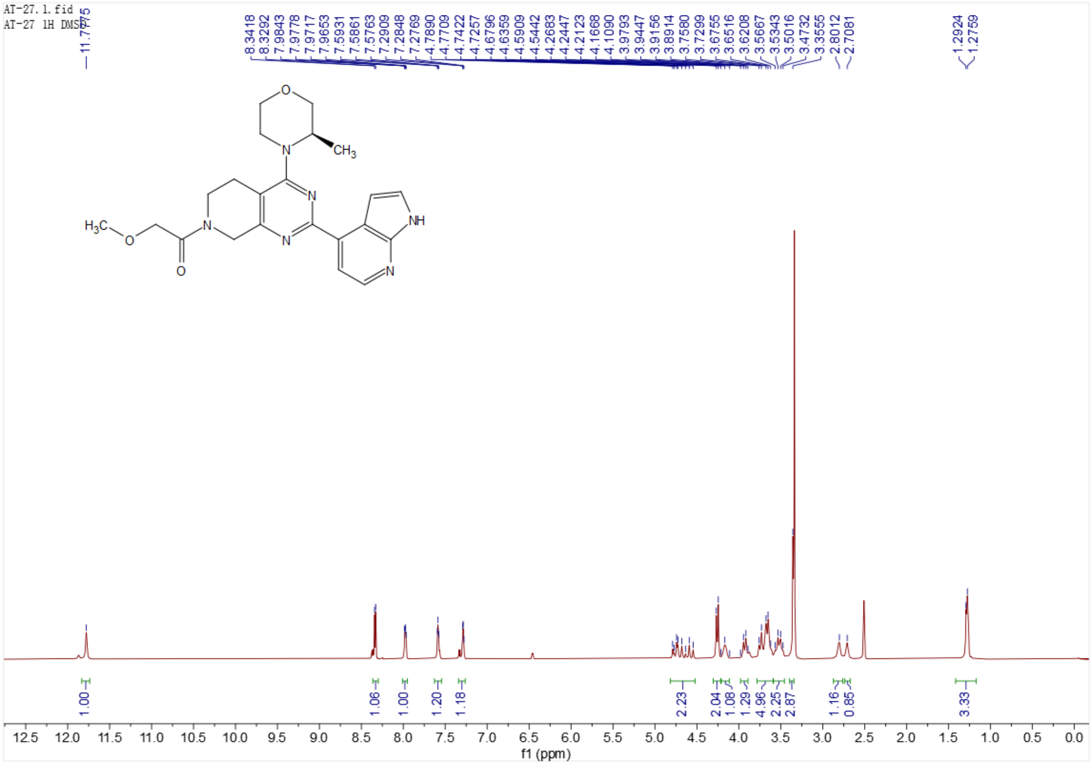

^{13}C-NMR of compound **ZH-7**

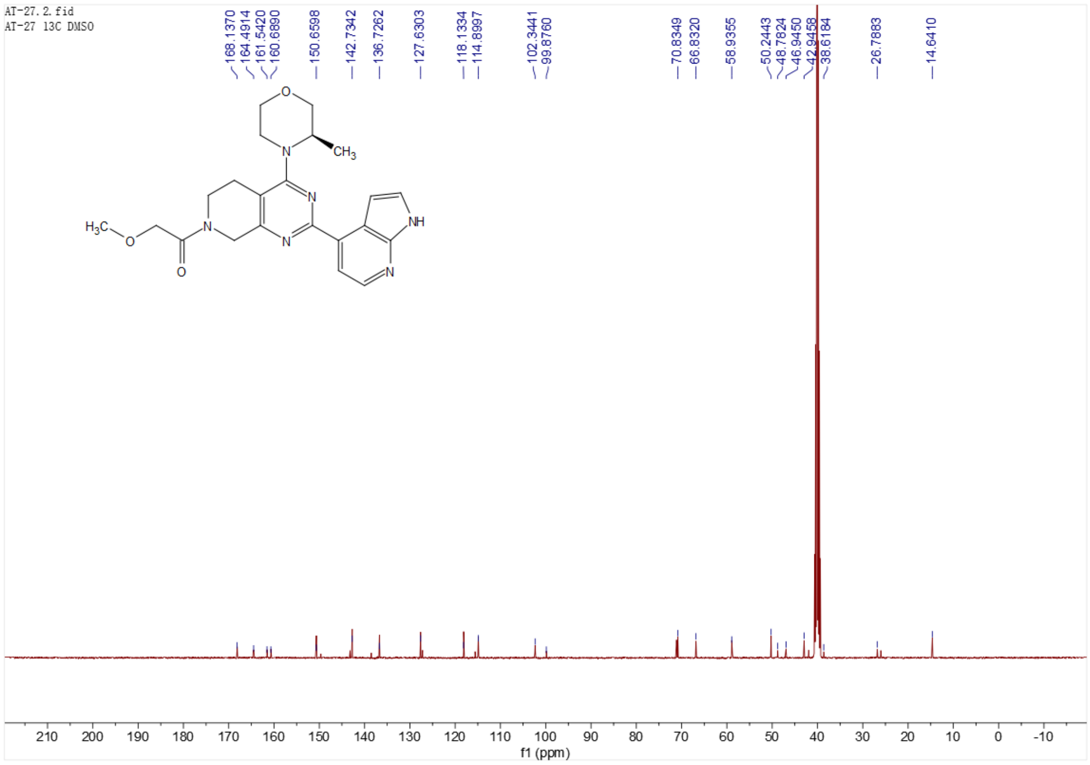

HRMS of compound **ZH-7**

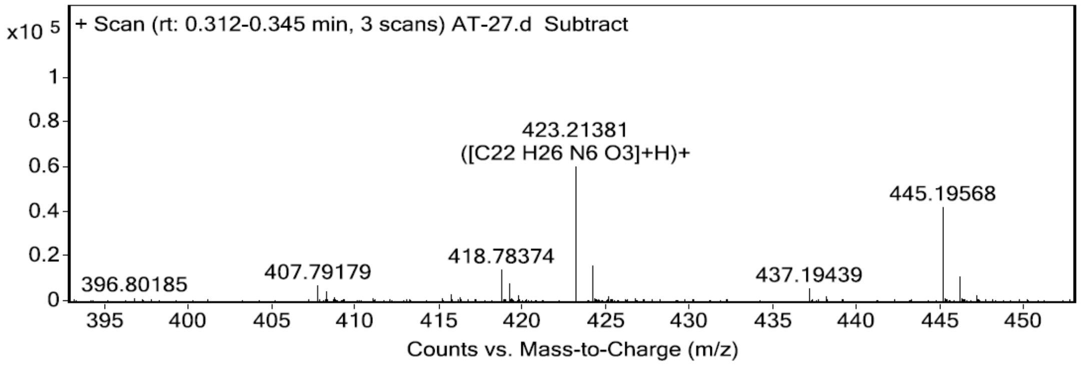

^{1}H-NMR of compound **ZH-8**

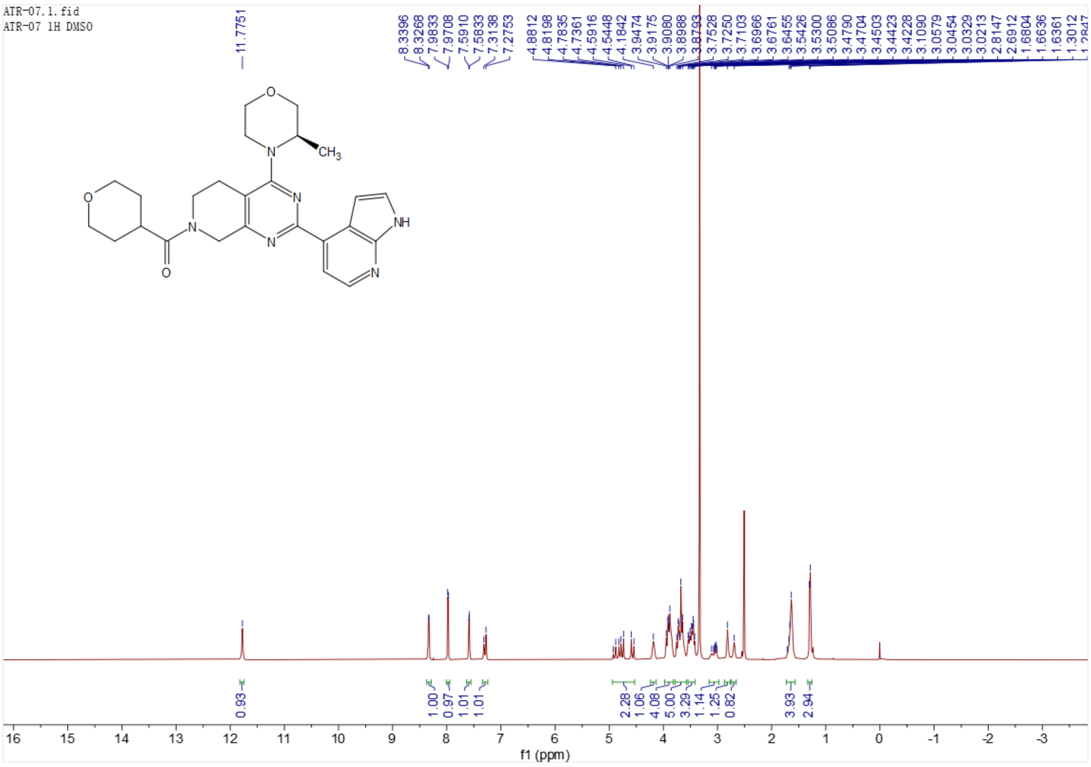

^{13}C-NMR of compound **ZH-8**

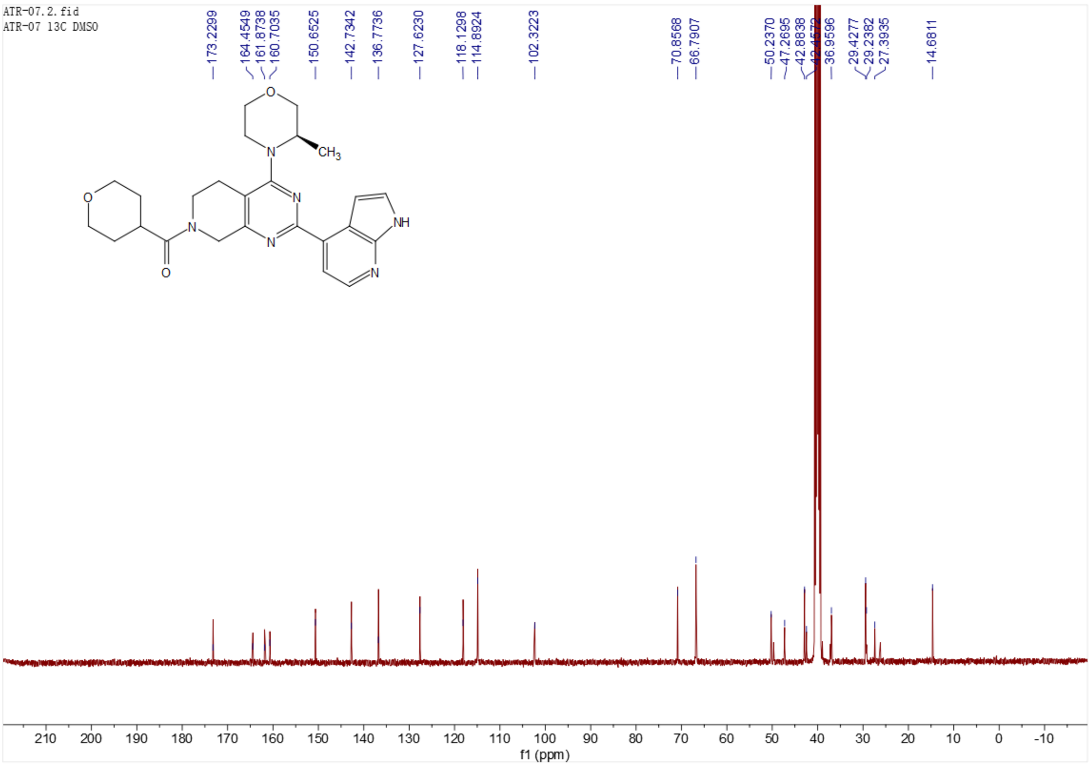

HRMS of compound **ZH-8**

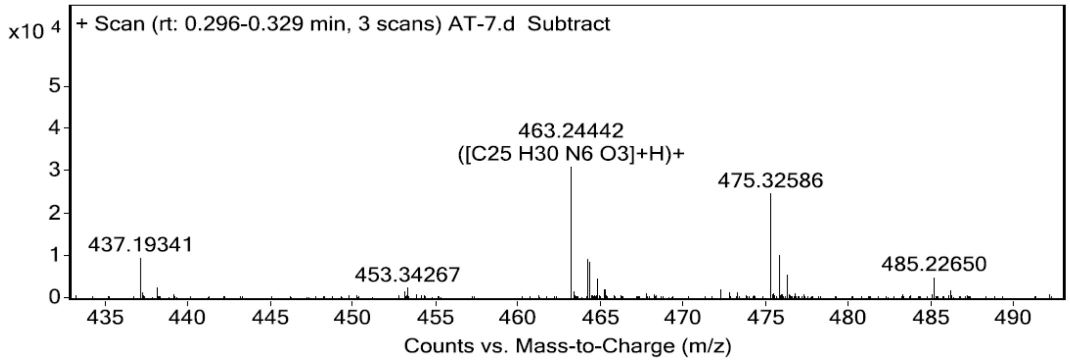

^{1}H-NMR of compound **ZH-9**

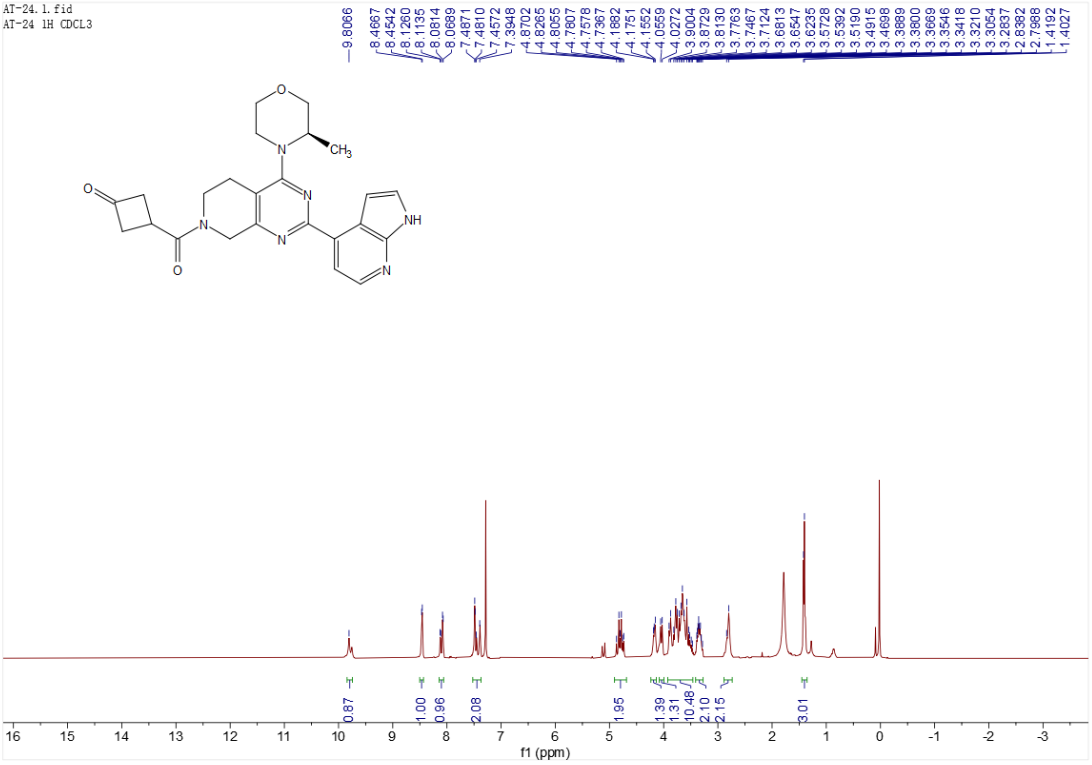

^{13}C-NMR of compound **ZH-9**

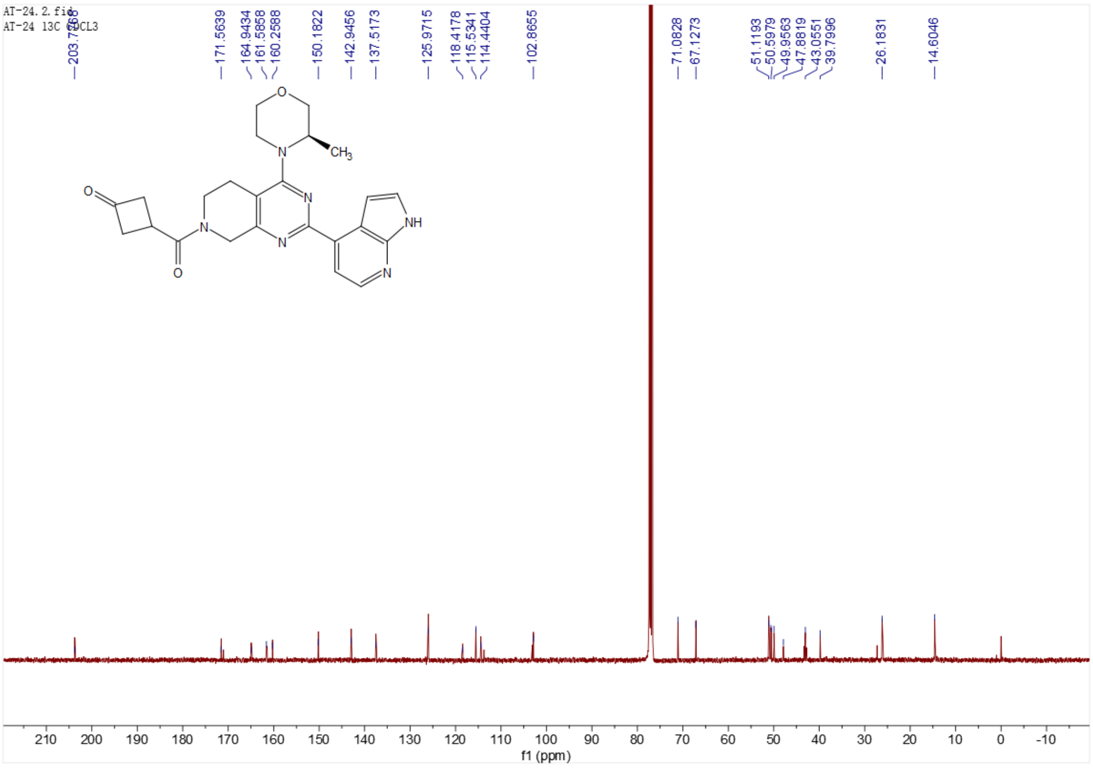

HRMS of compound **ZH-9**

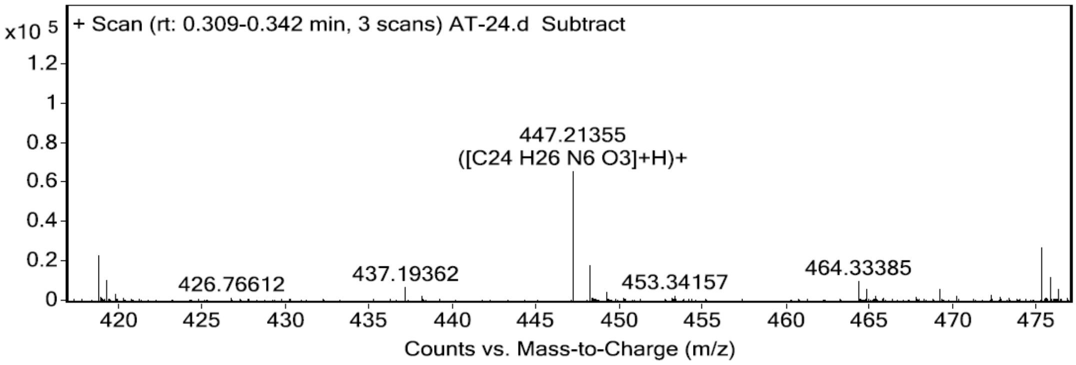

^{1}H-NMR of compound **ZH-10**

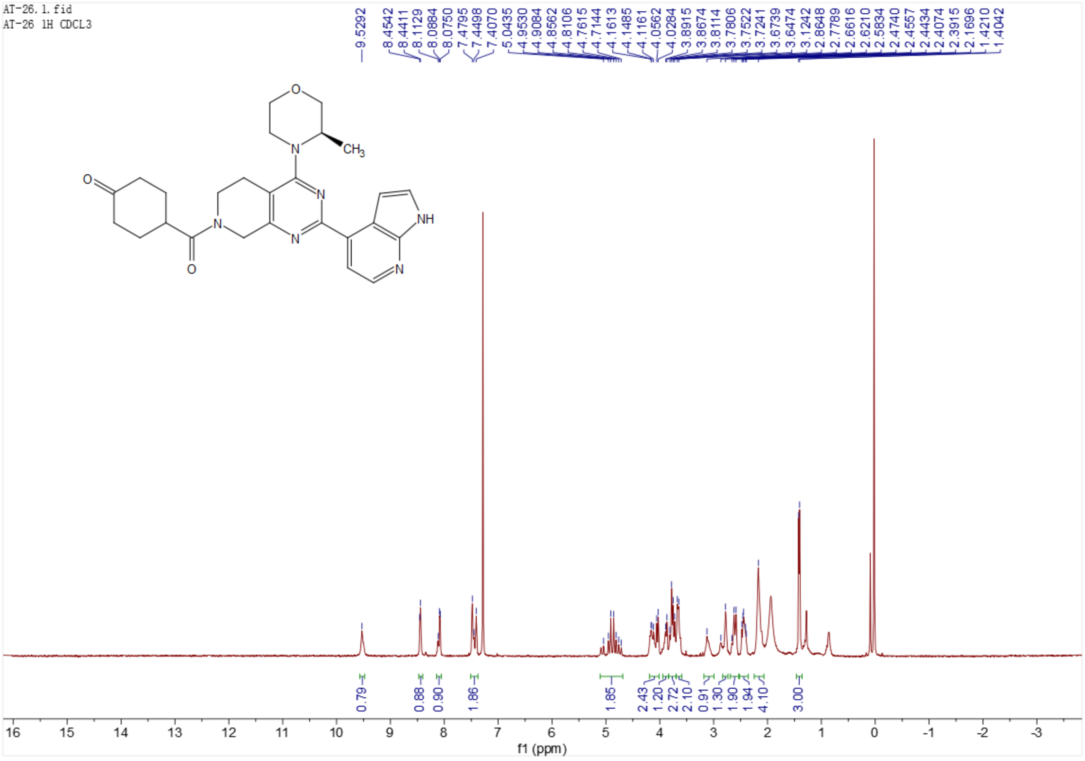

^{13}C-NMR of compound **ZH-10**

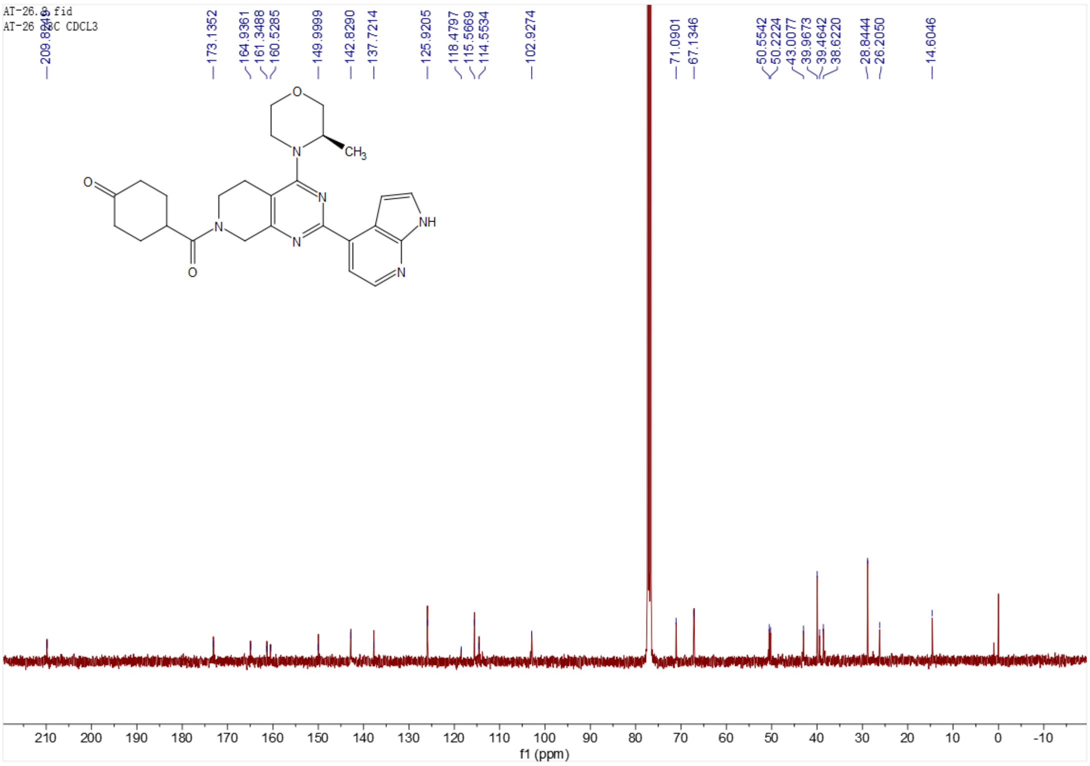

HRMS of compound **ZH-10**

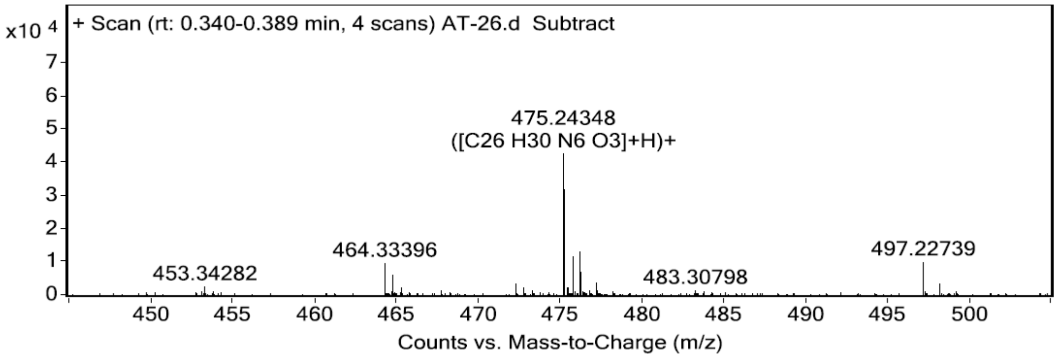

^{1}H-NMR of compound **ZH-11**

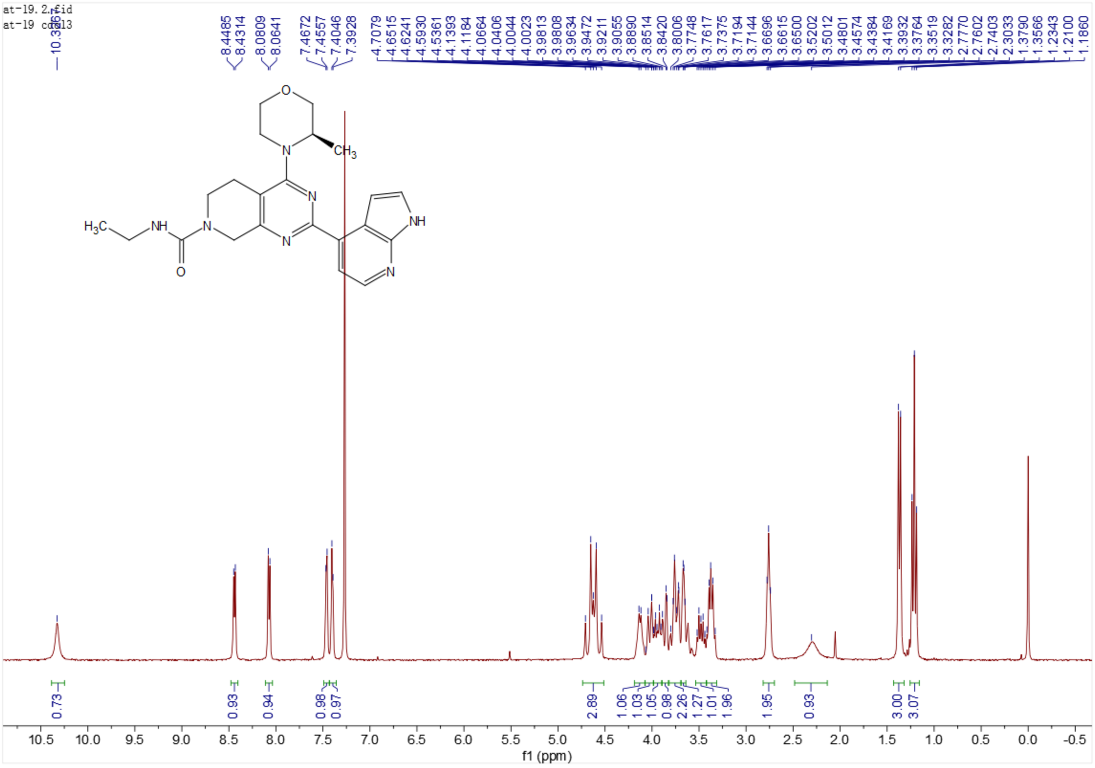

^{13}C-NMR of compound **ZH-11**

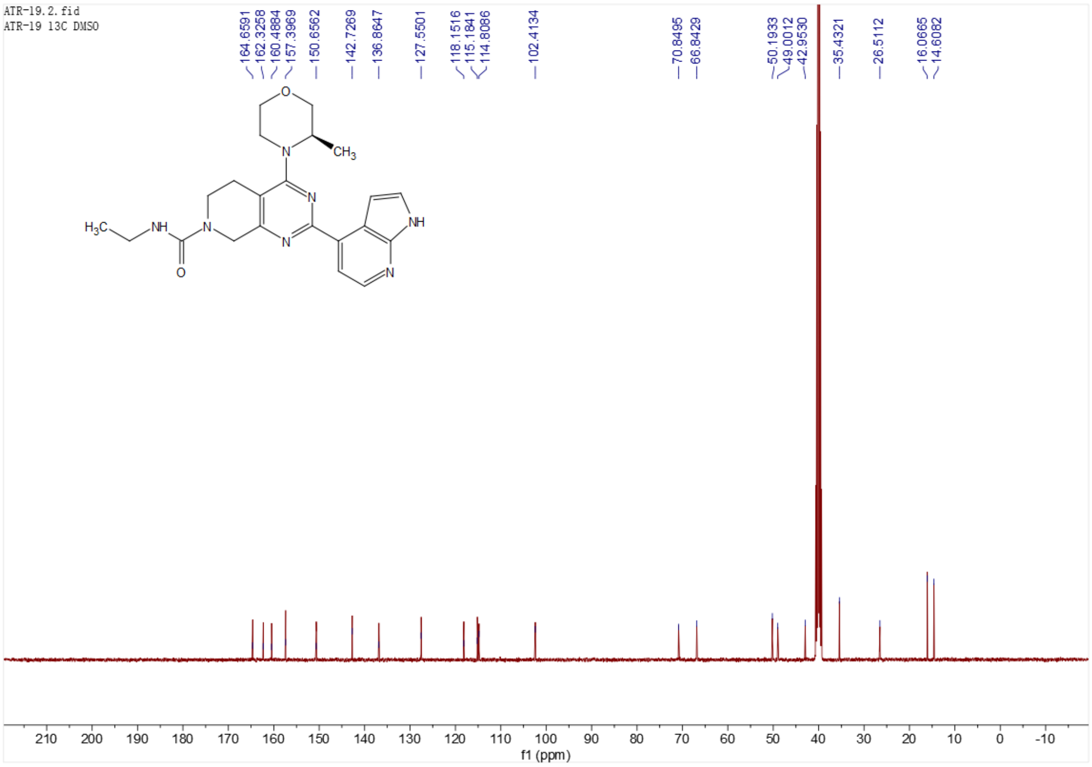

HRMS of compound **ZH-11**

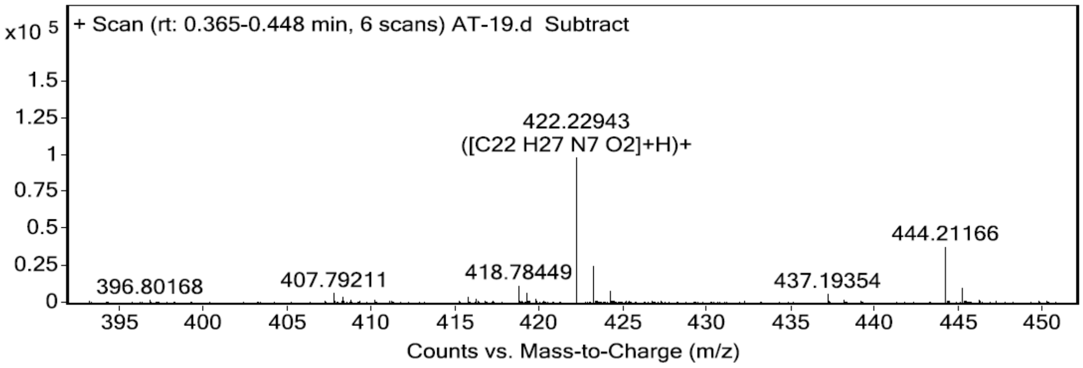

^{1}H-NMR of compound **ZH-12**

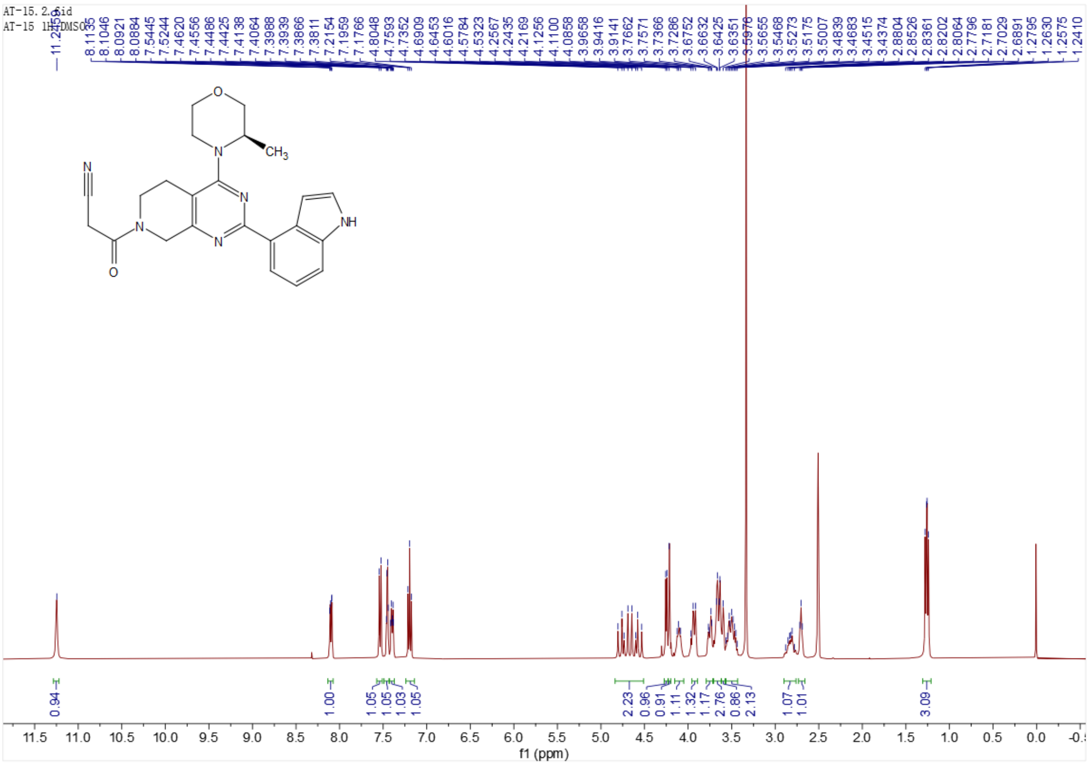

^{13}C-NMR of compound **ZH-12**

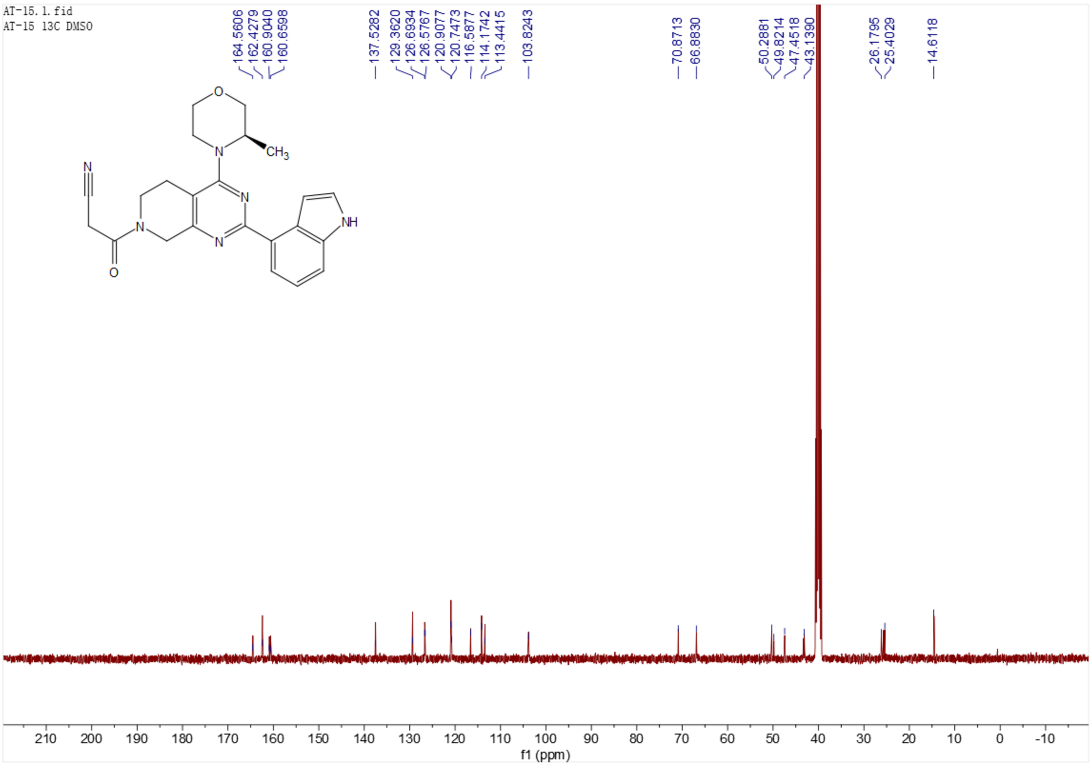

HRMS of compound **ZH-12**

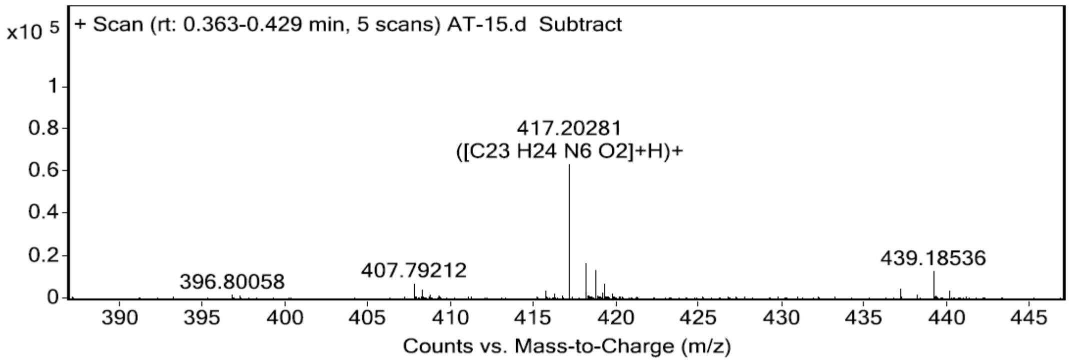

^{1}H-NMR of compound **ZH-13**

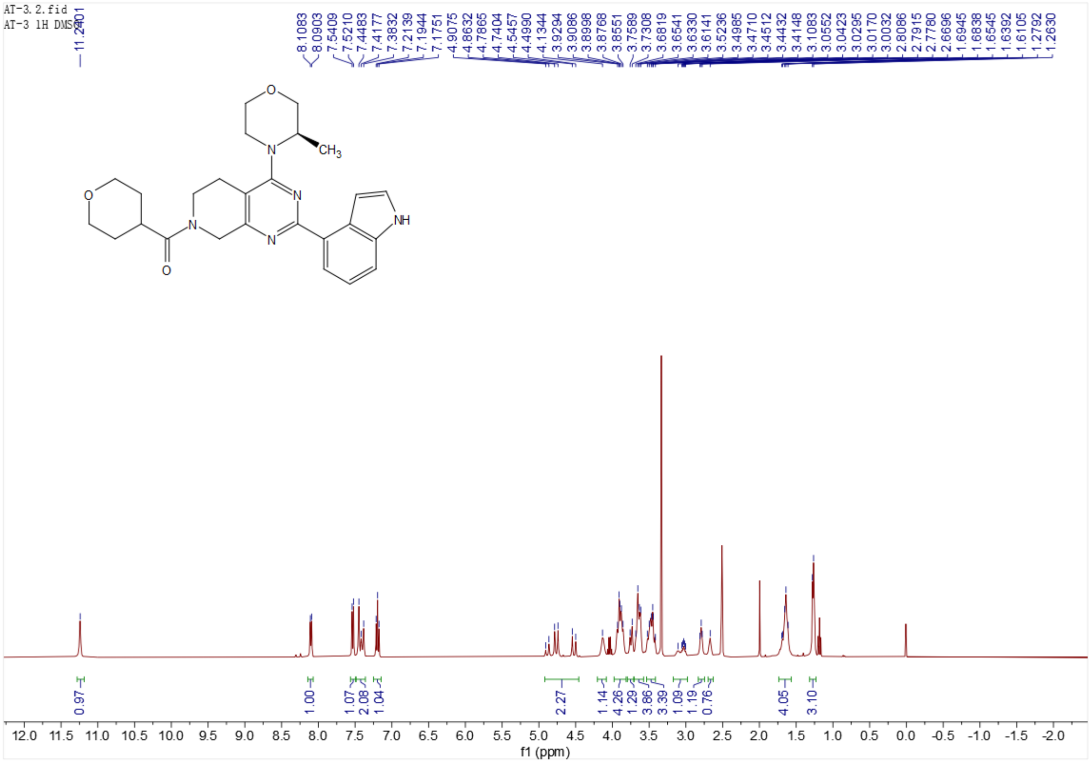

^{13}C-NMR of compound **ZH-13**

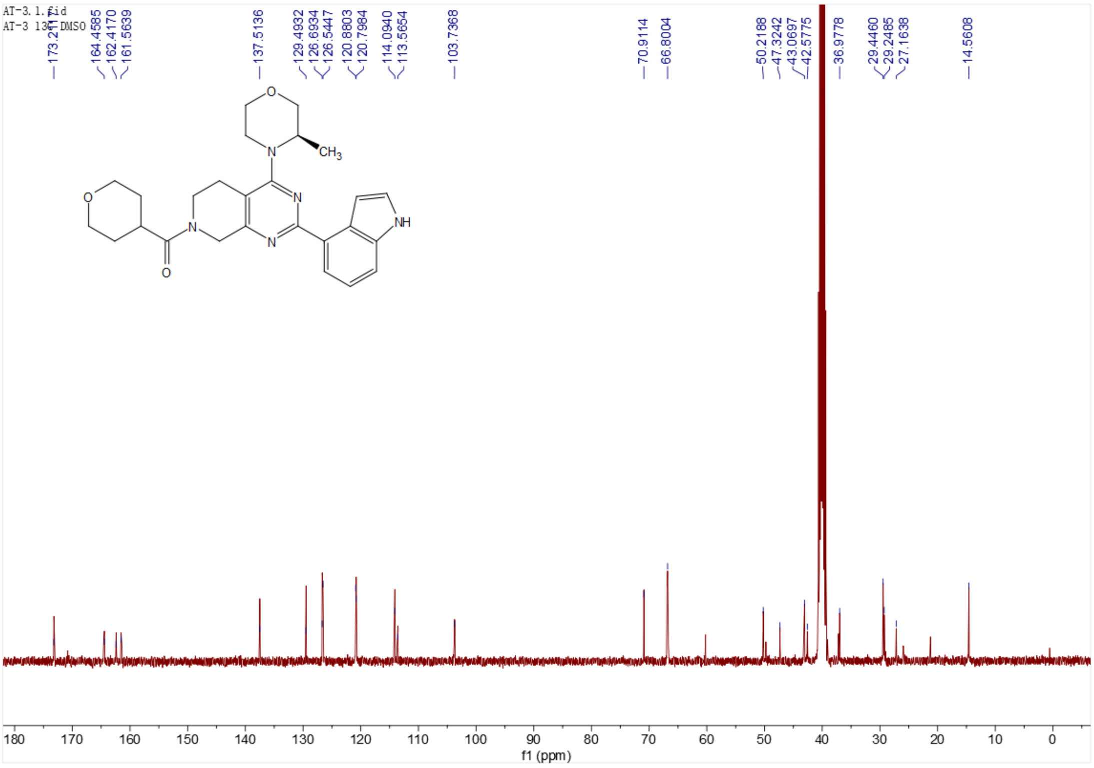

HRMS of compound **ZH-13**

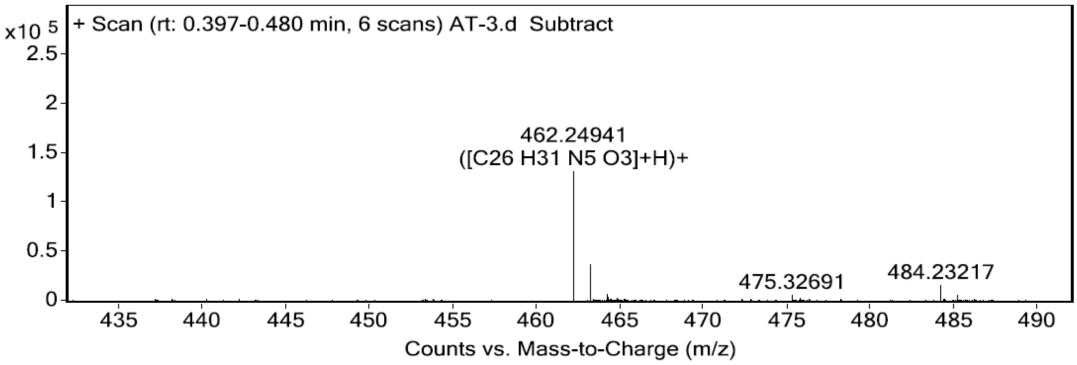

^{1}H-NMR of compound **ZH-14**

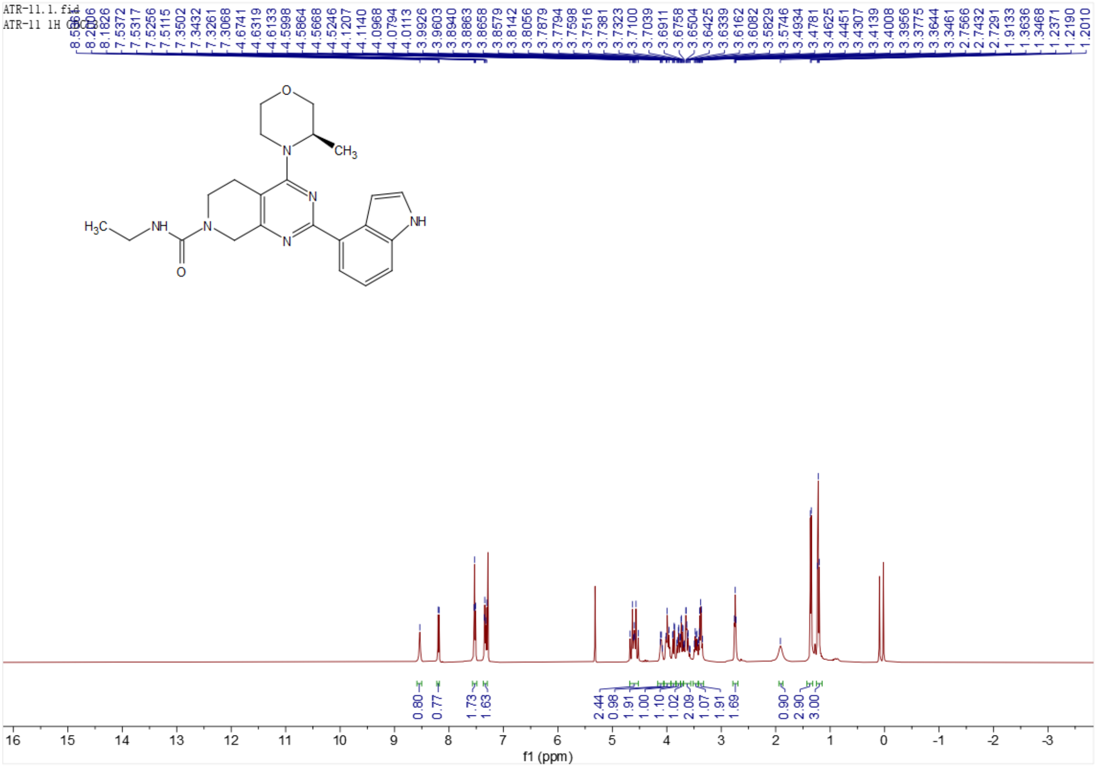

^{13}C-NMR of compound **ZH-14**

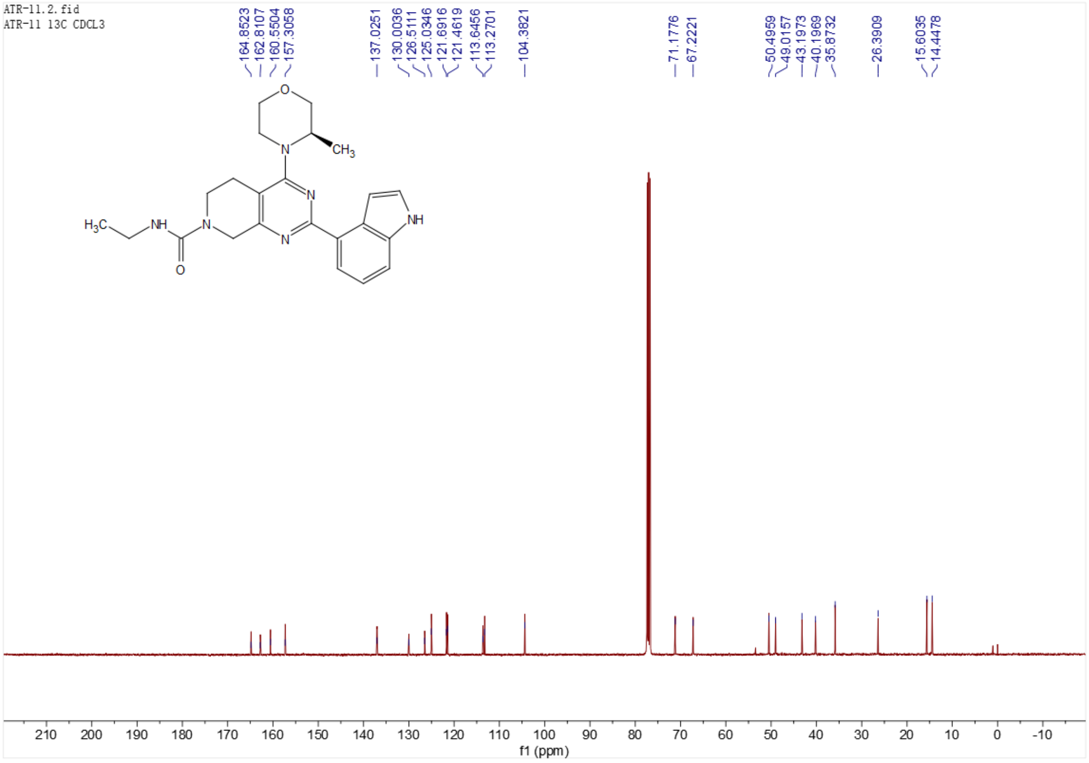

HRMS of compound **ZH-14**

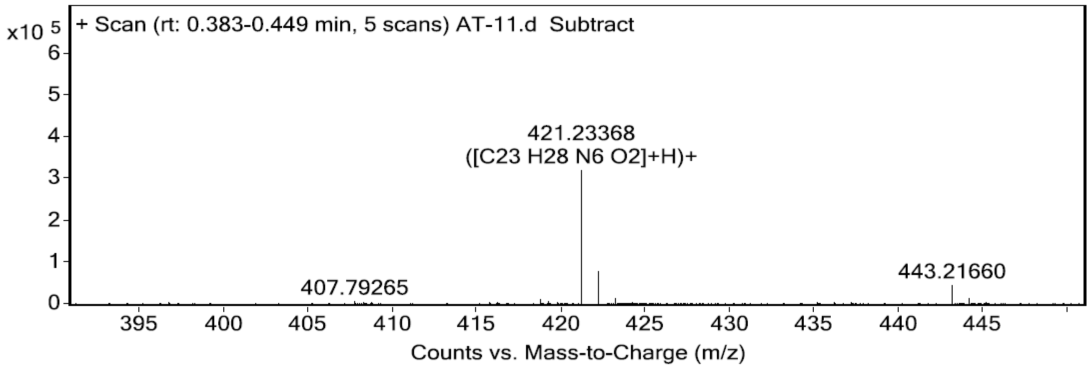

Supplement: Supplementary file 1 [file molecules-28-04521-s001.zip › molecules-2422245-supplementary.pdf]
